# *Per*- and polyfluoroalkyl substances in water and wastewater: A critical review of their global occurrence and distribution

**DOI:** 10.1016/j.scitotenv.2021.151003

**Published:** 2021-10-22

**Authors:** Sudarshan Kurwadkar, Jason Dane, Sushil R. Kanel, Mallikarjuna N. Nadagouda, Ryan W. Cawdrey, Balram Ambade, Garrett C. Struckhoff, Richard Wilkin

**Affiliations:** a Department of Civil and Environmental Engineering, California State University, 800 N. State College Blvd., Fullerton, CA 92831, USA; b Center for Environmental Solutions and Emergency Response, U.S. Environmental Protection Agency, 919 Kerr Research Drive, Ada, OK 74820, USA; c Department of Chemistry, Wright State University, 3640 Colonel Glen Highway, Dayton, OH 45435, USA; d Pegasus Technical Services, Inc., 46 E. Hollister Street, Cincinnati, OH 45219, USA; e Center for Environmental Solutions and Emergency Response, U.S. Environmental Protection Agency, 26 West Martin Luther King Drive, Cincinnati, OH 45268, USA; f Department of Chemistry, National Institute of Technology, Jamshedpur 831014, Jharkhand, India

**Keywords:** Perfluoroalkyl substances, Polyfluoroalkyl substances, PFAS, Environmental pollution, PFOS, PFOA

## Abstract

*Per*- and polyfluoroalkyl substances (PFAS) are a family of fluorinated organic compounds of anthropogenic origin. Due to their unique chemical properties, widespread production, environmental distribution, long-term persistence, bioaccumulative potential, and associated risks for human health, PFAS have been classified as persistent organic pollutants of significant concern. Scientific evidence from the last several decades suggests that their widespread occurrence in the environment correlates with adverse effects on human health and ecology. The presence of PFAS in the aquatic environment demonstrates a close link between the anthroposphere and the hydrological cycle, and concentrations of PFAS in surface and groundwater range in value along the ng L^−1^–μg L^−1^ scale. Here, we critically reviewed the research published in the last decade on the global occurrence and distribution of PFAS in the aquatic environment. Ours is the first paper to critically evaluate the occurrence of PFAS at the continental scale and the evolving global regulatory responses to manage and mitigate the adverse human health risks posed by PFAS. The review reports that PFAS are widespread despite being phased out—they have been detected in different continents irrespective of the level of industrial development. Their occurrence far from the potential sources suggests that long-range atmospheric transport is an important pathway of PFAS distribution. Recently, several studies have investigated the health impacts of PFAS exposure—they have been detected in biota, drinking water, food, air, and human serum. In response to the emerging information about PFAS toxicity, several countries have provided administrative guidelines for PFAS in water, including Canada, the United Kingdom, Sweden, Norway, Germany, and Australia. In the US, additional regulatory measures are under consideration. Further, many PFAS have now been listed as persistent organic pollutants. This comprehensive review provides crucial baseline information on the global occurrence, distribution, and regulatory framework of PFAS.

## Graphical Abstract

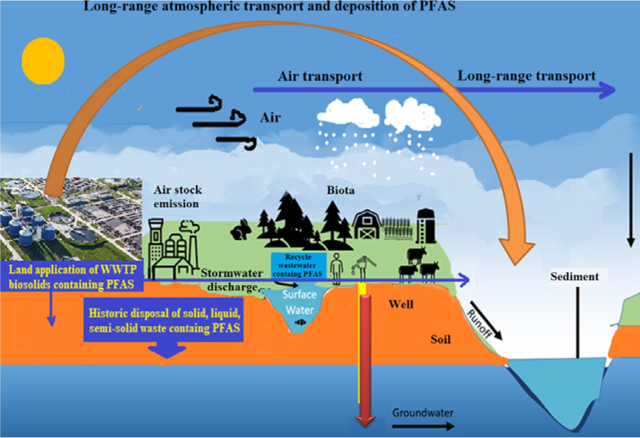

## 1. Introduction

### 1.1. Background

*Per*- and polyfluoroalkyl substances (PFAS) represent a diverse group of ≥ 4000 partially or fully fluorinated linear, branched, or cyclic compounds (Sunderland et al., 2019). They were first identified in the late 1940s and have become an integral part of industrial and consumer products worldwide. They are an active ingredient in adhesives, fire-fighting foams, cosmetics, paper products, and are used as stain and water repellants in the textile industry. PFAS are used in the manufacturing of aqueous film-forming foams (AFFFs) for firefighting operations (accounting for 1–5% *w*/w) semiconductors, lubricants, coating additives, surfactants, agricultural applications, pesticides, and they are used as erosion inhibitors in the aviation industry (Oliaei et al., 2013; Aminot et al., 2019; Sunderland et al., 2019; W. Zhang et al., 2021; Z. Zhang et al., 2021). Due to their hydrophobic and oleophobic properties, PFAS are also used in many household items such as cookware and food packaging. The highest number of PFAS and their precursors are used in the textile industry, followed by paper packaging and aftermarket consumer products (Glüge et al., 2020).

Most PFAS compounds have not been thoroughly evaluated, and their adverse impacts on human health or ecology have not been fully understood (Buck et al., 2011). PFAS exposure can occur through various pathways, but its severity depends on proximity to the exposure source, source concentration, and frequency of exposure. Numerous studies hsave documented the presence of PFAS in human blood samples, and in samples from terrestrial and aquatic flora and fauna (Quinete et al., 2009; Ahrens and Bundschuh, 2014; North Carolina Department of Health and Human Services, 2018; Gebbink and van Leeuwen, 2020). A comprehensive study confirmed the presence of perfluorooctanoic acid (PFOA) at concentrations of up to 147 ng L^−1^ in blood samples of residents living near a fluorochemical processing plant (FPP) in the Netherlands (Gebbink and van Leeuwen, 2020). Similarly, the North Carolina Department of Health and Human Services reported the presence of PFOA (0.4–7.3 μg L^−1^; median concentration = 1.75 μg L^−1^) and perfluorooctane sulfonic acid (PFOS) (1.4–34.6 μg L^−1^; median concentration = 5.4 μg L^−1^) in the blood serum of residents living near an FPP. PFAS exposure may result in decreased spleen and thymus weights and cellularity, reduced production of specific antibodies, reduced survival after influenza infection, immunosuppression, and altered cytokine production (Grandjean et al., 2012). PFAS have been associated with the onset of numerous other human ailments, including cancer, yet there are no restrictions on their use in consumer products and for industrial applications. Further, PFAS can easily undergo bioaccumulation due to their long persistence times. They are listed as emerging environmental contaminants (Li et al., 2010; Bai and Son, 2021).

PFAS pose a threat to flora and fauna, including aquatic species. Bioaccumulation of PFAS varies among species within the natural food web, making it difficult to accurately assess ecological risks due to the vast differences in species’ habitat, exposure, and bioaccumulation (Ahrens and Bundschuh, 2014). Furthermore, the magnitude of various PFAS accumulation differs between species’ internal organs. PFOS can accumulate in higher concentrations within the liver than within muscle tissues (liver>muscle>kidney), whereas the opposite may be true for PFOA (muscle>kidney>liver). Bioaccumulation also varies among aquatic species, with PFOA accumulating in higher concentrations in mussels versus fish (Quinete et al., 2009). Mean concentrations of PFOS up to 1900 ng g^−1^ have been reported in different aquatic species including various species of seals, porpoises, whales, and dolphins, as well as polar bears (Houde et al., 2011). The long-term persistence of PFAS in the environment could have impacts at multiple levels of the food web (Corsini et al., 2014; Ghisi et al., 2019; Goodrow et al., 2020).

Several review papers on PFAS occurrence, distribution, and exposure have been published in the last decades. For instance, (Rayne and Forest, 2009; Houde et al., 2011; Ahrens, 2011; Ahrens and Bundschuh, 2014; Rahman et al., 2014; Jian et al., 2017; Xiao, 2017; Sunderland et al., 2019; Vo et al., 2020; Schulz et al., 2020; Winchell et al., 2021), reviewed the occurrence, fate, effect, exposure of PFAS in the aquatic environment, as well as analytical methodologies in the field. Other researchers (Chen et al., 2009; Wang et al., 2015; Banzhaf et al., 2016; Q. Wang et al., 2020; S. Wang et al., 2020) have reviewed similar aspects of PFAS at regional levels. Additionally, (Parsons et al., 2008; Du et al., 2014; Rahman et al., 2014; Espana et al., 2015; Kucharzyk et al., 2017; Zhang et al., 2019; Mahinroosta and Senevirathna, 2020; Wanninayake, 2021; W. Zhang et al., 2021; Z. Zhang et al., 2021) have reviewed the remediation of PFAS in soil and aqueous environments, including focused reviews on adsorption, bioremediation, nanotechnology, novel remediation technologies, and the comparison between these technologies. These review papers each dealt with a specific aspect of PFAS, often focusing on a specific region, environmental medium, or remediation technology. None of these review papers provided comprehensive coverage of PFAS at the continental scale. Therefore, this study aimed to provide a comprehensive review of PFAS occurrence, distribution, regulation, and associated health risks. In this study, we critically review research published in the last decade on the global occurrence and distribution of PFAS in the aquatic environment. Furthermore, we evaluated the occurrence of novel PFAS, and the evolving global regulatory response to manage and mitigate the adverse human health risk posed by PFAS. In the process, we provide crucial information regarding the global occurrence, distribution, and regulatory framework of PFAS. This review is unique in its broad coverage as it does not focus on a specific region, or environmental medium. We aim to assist environmental professionals, researchers, and regulators worldwide by providing a baseline information on PFAS and emphasizing the need to manage and mitigate the emerging environmental crisis.

### 1.2. PFAS categories/nomenclature and environmental relevance

PFAS are a diverse class of chemicals with a common aliphatic carbon backbone in which hydrogen atoms are fully (perfluorinated) or partially (poly-fluorinated) replaced by fluorine. They possess high polarity, and strong, chemically inert carbon-fluorine (CF) bonds, which give them unique chemical attributes, including extremely high thermal and chemical stability (Rahman et al., 2014; Fernandez et al., 2016). These unique properties of PFAS make them highly stable, persistent, and recalcitrant chemicals, thereby making it difficult to manage their occurrence in various environmental media such as soil, water, and air. Numerous naming conventions exist for groups of PFAS; for simplicity, this paper uses a classification system that represents PFAS as aliphatic substances which contain −C_n_F_2n+1_ (*n* ≥ 1) moiety (Buck et al., 2012). They can be further categorized into long-chain acids, like perfluoroalkyl carboxylic acids (PFCAs, C_n_F_2n+1_COOH, *n* ≥ 7), perfluoroalkyl sulfonic acids (PFSAs, C_n_F_2n+1_SO_3_H, *n* ≥ 6), and their precursors; and short-chain acids (-C_n_F _2n+1_, *n* ≤ 6) (Yao et al., 2020). PFOS (C_8_F_17_SO_3_H) and PFOA (C_7_F_15_COOH) are among the most commonly used PFAS, and are the most frequently detected in the environment, and most researched of these compounds (Buck et al., 2011).

The global distribution of PFAS in aquatic environments, human tissues, and animal tissues varies significantly depending on geographic location, primarily due to their inherent chemical properties, as well as their production methods. For example, in a region where PFAS were predominantly produced via traditional electrochemical fluorination (ECF) – a method which was discontinued in the U.S. in 2011 and which produces branched isomers as a byproduct – will have a higher ratio of branched isomers as an environmental pollutant compared to the PFAS produced by fluorotelomerization (Schulz et al., 2020; Baabish et al., 2021; Sharifan et al., 2021). Furthermore, structural variation between PFAS affects their transport in the environment. Linear PFAS prefer to partition into the soil and sediments, whereas the branched isomers prefer to remain in aqueous phase. This difference in their environmental behavior can serve as an additional tool for tracing the source of PFAS, and their persistence times (ITRC, 2020; Schulz et al., 2020). Their chain length and functional moieties determine their environmental occurrence and distribution with hydrophilic short-chain PFAS (C ≤ 8) typically found in surface waters, whereas the hydrophobic long-chain PFAS (C > 8) tend to bioaccumulate in fish tissues and sediments (Goodrow et al., 2020; Bai and Son, 2021).

## 2. Review methodology

We examined multiple studies on PFAS sources, occurrence, distribution, and fate and transport. Compiling complete information was challenging because it is difficult to include all results regarding the concentrations and occurrences of multiple PFAS across various studies. Therefore, we prioritized our efforts and streamlined the review process and data collection by primarily focusing on the most common PFAS: PFOA and PFOS. Even though manufacturers voluntarily phased out these compounds, their continued occurrence remains a concern for global human and ecological health. In 2021, the United States Environmental Protection Agency (US EPA) decided to initiate the regulation of PFOS and PFOA through the National Primary Drinking Water Regulation process. As part of this process, the Science Advisory Board (SAB) - PFAS Review Panel proposed to hold public meetings to: 1) review and gather feedback on health effects to determine Maximum Contaminant Level Goals for PFOA and PFOS; 2) discuss potential reductions in health risk vis-à-vis reductions in drinking water concentrations; and 3) develop an approach to evaluate cumulative health risk due to exposure to PFAS mixtures (PFAS Strategic Roadmap, 2021; Federal Register, 2021). Through the Notice of Proposed Rulemaking, the EPA may designate PFOA and PFOS as hazardous substances under the Comprehensive Environmental Response, Compensation, and Liability Act (CERCLA) which will mandate that facilities report PFOA and PFOS releases. Such a designation will enable federal, state, and other authorities to access information about the magnitude of releases and seek recovery costs for cleanup operations (PFAS Strategic Roadmap, 2021). As such, this targeted review is intended to compile disparate information regarding these two extensively studied and frequently detected compounds into a cohesive narrative for global readers. Other novel PFAS are also briefly reviewed because they are now extensively used in many industrial applications in place of legacy PFAS.

We gathered information from academic sources, as well as reputable non-academic sources, including federal, state, and local government records, and reports obtained from agency websites. Given the vast amount of information currently available, our literature search was specifically focused on studies published since 2000. We conducted extensive searches of keywords individually and in combination to procure the relevant publications. Our search included the following terms: PFAS, PFOA, PFOS, Perfluorochemicals (PFCs), Occurrence, Distribution, Persistent Organic Pollutants (POPs), Emerging Contaminants, C8, Environment, Perfluoroalkyl substances, polyfluoroalkyl substances, and POPs. This yielded numerous studies ranging from a regional to global scale. Studies were screened based on their relevance, rigor, and substance of focus, and not all studies were included in the review. We mainly reviewed large-scale studies that had analyzed multiple samples from various media across multiple countries.

## 3. Sources and occurrence of PFAS in the environment

The release of PFAS into the environment occurs through two major sources. Point sources are discrete and stationary, such as FPP, other industrial/manufacturing facilities, firefighting training sites, wastewater treatment plants (WWTPs), and landfills. Nonpoint sources are diffuse sources of unknown origin/location and often involve the release from multiple sources, and include atmospheric transport of volatile PFAS, biological and chemical breakdown of precursor compounds into stable PFAS, surface runoff, precipitation, and consumer product breakdown (Benskin et al., 2012; Wang et al., 2015; Ruyle et al., 2021).

WWTPs are among the most thoroughly studied point sources of PFAS in surface waters. Wastewater, as well as biosolids, and recycled wastewater can serve as perennial sources of PFAS in the environment. The application of WWTP biosolids and recycled wastewater often causes PFAS contamination in surface and well water (Lindstrom et al., 2011; Szabo et al., 2018); and while many promote the use of recycled water for drinking water and irrigation purposes, it is important to recognize that treated effluent often contains numerous micro-pollutants, including PFAS, that may impact human health, and surface water and groundwater quality (Plumlee et al., 2008; Díaz-Cruz et al., 2009; Xiao, 2017; Szabo et al., 2018). Another primary point source of PFAS contamination in surface water and groundwater is the AFFFs used in firefighting training exercises, and PFAS contamination in water due to AFFFs has been widely documented, especially in the USA. The US military has historically been the largest consumer of AFFF, and specific sites where AFFFs were traditionally used include military bases and airports (Moody and Field, 2020). The US Air Force has identified approximately 200 installations in the USA where PFAS-containing AFFFs have been used. Furthermore, a cyclic perfluorinated acid (PFA) such as perfluoroethylcyclohexanesulfonate (PFECHS), was used as an erosion inhibitor in aircraft hydraulic fluids, which is as recalcitrant to environmental degradation as aliphatic PFAS (Stefanac et al., 2018). In 2011, PFECHS was reported for the first time in predatory fish (<Method Detection Limit (MDL) to 3.7 ng g^−1^ wet weight in whole-body homogenate) in the Great Lakes, and surface waters of the lake (0.16–5.7 ng L^−1^) (Silva et al., 2011).

PFAS are utilized extensively in industrial/manufacturing activities, including in the manufacturing of packaging, consumer goods, textiles, firefighting foams, aviation hydraulic fluid, semiconductors, pesticides, electronics, and protective non-stick coatings. Waste originating from these activities often contains low levels of PFAS and is typically managed through landfilling, wastewater treatment, and incineration. However, none of these approaches result in the complete breakdown or degradation of PFAS, but only isolate or transfer them from one environmental matrix to another (Stoiber et al., 2020). Perfluorinated PFAS, which have all the carbon atoms fully fluorinated are very stable and not easily degraded. In contrast, polyfluorinated substances contain carbon atoms that are partially fluorinated, making them less stable and more likely to degrade at least to a certain extent. They eventually form relatively stable perfluorinated moieties, which are typically referred to as precursor compounds (Göckener et al., 2021).

Recent advancements in analytical chemistry made it possible for researchers to detect and quantify PFAS at extremely low concentration levels (pg L^−1^). These advances in the analytical detection of certain PFAS in the environment has prompted the global monitoring of PFAS (Ahrens, 2011). Since 2010, some limited progress has been made toward implementing a global monitoring program under the Stockholm Convention on Persistent Organic Pollutants. An extensive global assessment of PFOS in water has been conducted through the second phase of the Stockholm Convention’s Global Monitoring Program. In 2009, PFOS was listed in Annex B, and in 2019 PFOA was listed in Annex A of the Stockholm Convention on Persistent Organic Pollutants. In contrast, other analogues such as perfluorohexane sulfonic acid (PFHxS) and PFCA are currently being reviewed as candidates for listing under the convention (UNEP, 2019; Barceló and Ruan, 2019). Typical background concentrations of PFAS in areas not directly impacted by point sources may only be detectable in pg L^−1^ or ng L^−1^ (Ahrens, 2011). For example, PFAS were detected in low to mid concentrations (pg L^−1^ and pg m^−3^) in remote Canadian Arctic snow, air, and atmospheric particles (Young et al., 2007; Stock et al., 2007). In most samples from aqueous environments, PFAS concentrations range from Non-detect (ND) - low ng L^−1−^. Detection of PFAS at low concentrations is critical because even exposure at low concentrations can have adverse effects on human health (Dean et al., 2020). Epidemiological studies have shown that some PFAS can cause reproductive and developmental defects even at low concentrations (USEPA, 2017; Tarapore and Ouyang, 2021). The US EPA health-based advisory concentration is 70 ng L^−1^, whereas some states in the USA, such as Vermont, have set the concentration values at 20 ng L^−1^. Table 1 shows the updated health-based standards for drinking water in the United States and other countries.

Due to the high water solubility and persistence of some PFAS, ocean, groundwater, and surface waters are the major global sinks for these compounds. The presence of PFAS in waste streams affects surface and groundwater, the two primary sources of drinking water worldwide. Between 2004 and 2010, the analysis of surface water samples from 41 cities in 15 countries revealed the presence of PFOS and PFOA in all samples with average concentrations ranging from non-detect ND – 70.1 ng L^−1^, and 0.2–1630.2 ng L^−1^, respectively (Kunacheva et al., 2012). In this study, rivers in the UK tested positive for PFAS with maximum mean concentrations of PFOS at 19 ng L^−1^, whereas in Osaka, Japan, surface waters had maximum concentrations of PFOA at 1630.2 ng L^−1^. Average PFOA concentrations were generally below 100 ng L^−1^ in other cities included in the study area. In Eastern Spain, PFAS were detected in water, sediments, and biota from the Jucar River with concentrations ranging from 0.04–83.1 ng L^−1^, 0.22–11.5 ng g^−1^, and 0.63–274 μg kg^−1^, respectively. The higher concentration of PFAS in biota compared to water, indicates a potential bioaccumulation pathway (Campo et al., 2016).

The widespread occurrence of PFAS in natural waters denoted above increases the possibility of exposure via drinking water. Jian et al. (2017) reviewed the occurrence of PFCs in various water samples in fourteen countries (Australia, Brazil, China, Faroe Islands, France, Germany, Ghana, Japan, Korea, Netherlands, Norway, Spain, Thailand, and USA) and found that, in most countries the concentration of PFOA ranged from 0 to 20 ng L^−1^ with the highest concentration in tap water and well water samples from Japan (110 ng L^−1^) and Ghana (800 ng L^−1^) respectively, whereas the highest PFOS concentrations were detected in tap water collected from Ghana (100 ng L^−1^) and Spain (40 ng L^−1^). They found that among the types of drinking water samples PFAS levels were highest in well water followed by tap water, bottled water, drinking water, and raw water (Jian et al., 2017). The ingestion of PFAS through drinking water is a definite exposure pathway for the human population that relies on PFAS-contaminated water. However, our understanding of PFAS occurrence, and the human health and ecological effects associated with chronic exposure at low concentrations, has only improved over the last two decades.

To better understand the occurrence of these compounds, it would be helpful to understand how PFOA and PFOS compare with other carcinogens such as the aromatic compound benzene. The comparison is made to highlight their relative occurrence and mobility in the subsurface environment. The emphasis will be on chemical make-up of the PFAS (Table S1) to demonstrate their relative persistence compared to benzene.

The molecular weights of PFOA and PFOS are higher than that of benzene by 5.3 and 6.4 times, respectively. The boiling points of PFOA and PFOS are higher than that of benzene by 2.4 and 3.2 times, respectively, whereas their respective vapor pressures are 181 and 4760 times lower than that of benzene. The log Koc of PFOA and PFOS is similar to that of benzene. The water-solubility of PFOA is 5.3 times higher, whereas that of PFOS is 2.6 times lower compared to those of benzene. Based on groundwater monitoring conducted by the United States Geological Survey (USGS) and data obtained from the Unregulated Contaminant Monitoring Rule (UCMR) program, benzene was detected in 1.3% of public water supply wells between 1993 and 2007, whereas PFOA/PFOS was detected in 1% of groundwater samples monitored between 2013 and 2015 (Newell et al., 2020). The higher detection rate of benzene is attributed to the greater use and environmental discharge of benzene. The retardation factor (R) determines the relative mobility of compounds in the subsurface environment, and benzene (*R* = 1.305) and PFOA (*R* = 1.5) move at a similar rate, whereas PFOS (*R* = 6) moves nearly four times slower (Newell et al., 2020). These differences in chemical properties demonstrate the relative mobility of PFOA and PFOS compared to benzene.

## 4. Evolving regulatory response to PFAS

Over the last three decades, growing evidence of widespread contamination of water resources, human exposure, and the potential toxicity of PFAS has prompted action from regulatory bodies worldwide. Despite being first synthesized back in 1940s, PFAS have only recently been designated as contaminants of emerging concern (Aminot et al., 2019). Considerable research regarding the toxicity of these chemicals has been conducted by the chemical industry throughout the last half-century. The earliest studies primarily focused on factory workers and the potential toxic effects associated with PFAS exposure. For example, in 2005, DuPont funded a comprehensive, long-term epidemiological study focused on the health effects of PFOA in which they examined medical monitoring data of a cohort of over 30,000 affected residents and factory workers between 2005 and 2013. This study demonstrated a link between PFOA exposure and adverse human health conditions, including high cholesterol, ulcerative colitis, thyroid disease, testicular cancer, kidney cancer, and pregnancy-induced hypertension (Nicole, 2013; Winquist et al., 2013). The 3M Company, a primary manufacturer of PFOS in the USA, voluntarily phased out production in 2000 through a stewardship program in collaboration with the US EPA. In 2006, eight other significant manufacturers also agreed to phase out PFOA, although some still make limited use of it for specialty productions.

Recently, concerns were raised regarding human health and the occurrence of perfluoroalkyl sulfonates and carboxylates in various environmental matrices and trophic levels (Barceló and Ruan, 2019). Concentration limits were recently set for certain PFAS in European and North American countries. In 2016, the US EPA published an individual and combined Lifetime Health Advisory (LHA) level of 70 parts per trillion for PFOS and PFOA in drinking water (USEPA, 2016). Many states in the USA have established their health-based guidelines for PFOS, PFOA, and other PFAS in drinking water; including Delaware, Maine, Michigan, Minnesota, New Jersey, Nebraska, North Carolina, Pennsylvania, Texas, and Vermont (ASTSWMO, 2015; USEPA, 2017). Several countries have published administrative guidelines for levels of PFAS in water, including Canada, the United Kingdom, Sweden, Norway, Germany, and Australia (Cheremisinoff, 2017) (Table 1).

In the USA, comprehensive nationwide surveys have been conducted to monitor PFAS in important drinking water systems through the US EPA’s third UCMR Program. Every five years, the Safe Drinking Water Act requires the US EPA to publish a list of 30 contaminants that are currently not regulated but are known or most likely to occur in public water systems. These selected contaminants are included in a contaminant candidate list (CCL). Through the UCMR, all large water systems serving more than 10,000 people and a select group of 800 small water systems are tested for contaminants on the CCL. Six PFAS, including PFOA and PFOS, were monitored during UCMR 3, which was conducted from 2013 to 2015. Data from the UCMR 3 survey indicated that 4% of public water systems tested positive for at least one PFAS with 1.3% of the water systems exceeded the US EPA’s LHA threshold of 70 ng L^-1^. The same survey also reported that PFOA and PFOS concentrations in 0.3% and 0.8% of public water systems also exceeded the LHA (USEPA, 2016; Crone et al., 2019). The US EPA action plan published in February 2020 outlined the need to establish MCLs for PFOS and PFOA in drinking water; to determine whether these compounds should be declared as hazardous substances, and the resulting need of determining clean-up criteria for groundwater remediation (EPA, 2020). The European Commission (EC) has declared PFAS as emerging organic contaminants, and PFOS and its derivative as priority hazardous substances. In 2012, through its Water Framework Directive, the EC established a threshold concentration for PFOS in drinking water and fish for environmental quality assessment; and in 2013, it established Environmental Quality Standards against which to measure PFOS concentrations in inland surface waters and biota. The maximum allowable concentration is 36 μg kg^−1^ for inland surface waters, and 9.1 μg kg^−1^ for biota; while the mean annual concentration limit is 0.65 ng L^−1^ for inland surface waters (Pignotti et al., 2017).

Recently, the US Senate passed further legislation to regulate PFAS. The resolution requires that as of October 1, 2023, the Department of Defense must stop using AFFFs containing PFAS. The current LHA limit of 70 ng L^−1^ for PFOA and PFOS in drinking water does not have an adequate enforcement mechanism, and besides, manufacturers have already phased out the use of these compounds (Dean et al., 2020). Compounds such as GenX chemicals and short-chain PFAS are being manufactured as replacements for PFOS and PFOA; however, their toxicities, bioaccumulation behaviors, persistence, and interaction with the environment are still unknown. Exposure to short-chain PFAS – such as perfluorohexanoic acid (PFHxA) and related fluorotelomer precursors – reportedly has negligible risks to human health based on the current environmental levels of these compounds (Anderson et al., 2019). Given the lack of knowledge regarding the adverse effects of short-chain PFAS exposure, regulatory agencies worldwide should develop regulations, define PFAS as a class as opposed to individual compounds, and restrict the use of PFAS to critical applications while incentivizing their phasing out (Dean et al., 2020). Additionally, considerable uncertainty surrounds the monitoring of PFAS due to various factors, including the choice of sampling locations and variation in sources. These chemicals are not currently regulated due to the lack of robust, reliable, and reproducible data.

## 5. Worldwide distribution of PFAS

PFAS are among the largest group of chemicals widely used in industrial and commercial products and have been frequently detected in environments worldwide in drinking water, surface water, and groundwater across industrialized and developing countries. They have been widely detected in biota, ocean water, rainwater, snow, food, dust, air, and human serum. Analysis of drinking water samples collected from the United States, Canada, Burkina Faso, Chile, Ivory Coast, France, Japan, Mexico, and Norway indicate that three PFAS (PFCAs, PFSAs, and perfluoroalkyl acid precursors) were frequently detected with concentrations ranging from below detection limits to 39 ng L^−1^ (Kaboré et al., 2018). The National Health and Nutrition Examination Survey conducted in the USA, reported trace levels of PFAS in 98% of the human serum samples collected between 2003 and 2004 (Calafat et al., 2007; Kaboré et al., 2018). In this section, we review the occurrence of PFAS across five continents – Asia, North America, South America, Europe, and Africa – and discuss their occurrences in a variety of aqueous and terrestrial environments.

### 5.1. The occurrence of PFAS in the Asia-Pacific region

In its Second Global Monitoring Report on Persistent Organic Pollutants (2017), the Stockholm Convention reported that since 2009, PFOS were detected with increased regularity in various environmental matrices in the Asia-Pacific region. The report documented large-scale PFAS contamination in this region. For example, PFOS concentrations in the surface waters of China, Japan, South Korea, Philippines, and Thailand ranged from ND – 47 ng L^−1^, 0.02–230 ng L^−1^, 0.12–33 ng L^−1^, 0.39–4.2 ng L^−1^, and ND – 54 ng L^−1^, respectively (UNEP, 2017). Variation in the distribution of PFAS in this region is attributed to significant data variability, degree of industrialization and urbanization, choice of sampling locations, and analytical capabilities. Recent data on the distribution of PFOA and PFOS in China shows that the concentrations of both substances in environmental media were relatively low compared to those of other industrialized nations in the region (Baabish et al., 2021). In contrast, other studies have shown that high PFOS and PFOA concentrations were detected in Huangpu River, Yangtze River estuary, and Pearl River Delta located in China’s southern and eastern regions, and in rivers near industrial cities in several regions of China (So et al., 2007; Chen et al., 2009; Pan et al., 2018). Most studies attributed the distribution of PFAS to spatial variables, whereas some focused on the influence of PFAS characteristics on their distribution. Additionally, greater levels of ionic PFAS were found in north China, while neutral PFAS were more prevalent in south China. Q. Wang et al. (2020) showed that the PFOA and 8:2 fluorotelomer alcohol in outdoor air were predominantly made up of ionic and neutral species of PFAS, respectively. Ionic PFAS are more soluble and tend to accumulate in water, sediment, and soil, whereas neutral PFAS are generally more volatile and are commonly found in the atmosphere. Neutral PFAS can be converted to ionic PFAS via photochemical and biological processes; thus, the presence of ionic/neutral PFAS in leaves and tree bark could potentially be used as bioindicators for the presence of atmospheric PFAS (Q. Wang et al., 2020; S. Wang et al., 2020).

Given the widespread occurrence of PFAS in China, there is a high likelihood of exposure to residents living in adjacent areas. Between 1980 and 2000, the concentrations of PFAS in blood samples of Chinese citizens increased due to the ingestion of PFAS-contaminated drinking water (Chen et al., 2009; Wang et al., 2015). People living in eastern cities exhibited higher concentrations and frequency of detection compared to those of people living in western cities, and PFAS (PFOS and PFOA) concentrations were higher in southern and eastern China than in other areas of China. The highest PFOS and PFOA concentrations were detected in the Huangpu River and were 20.5 ng L^−1^ and 1590 ng L^−1^, respectively. Interestingly, Dongguan is the only city in China where PFOS concentrations in surface water exceeded the water quality criterion. Both Huangpu River and Dongguan are located in the area with extensive manufacturing of electronics, plastics, and textiles (Chen et al., 2009). In general, the concentrations of PFAS in aquatic systems were higher in more industrialized and urbanized areas than those in the less populated and more remote regions in China, indicating that their emission and distribution are closely related to regional urbanization and industrialization.

Lu et al. (2015) found significant variation in relative concentrations of 17 PFAS in surface water samples from rivers, lakes, and reservoirs in the Shanghai, Jiangsu, and Zhejiang Provinces in eastern China. In all three provinces, PFOA was detected at higher concentrations than PFOS especially in samples from Zhejiang Province, where the PFOA concentration range was 0.29–200 ng L^−1^ and that of PFOS was ND – 5 ng L^−1^ (Lu et al., 2015). The Huangpu river, a downstream tributary of the Yangtze river which flows through Shanghai Province and links Lake Taihu to eastern China, was found to contain PFOA (105 ng L^−1^) and PFOS (5.4 ng L^−1^) (Lu et al., 2015). Further, PFOA was the most prevalent PFAS in samples collected from all provinces, whereas PFHxA was the most prevalent in Zhejiang province. The distinctive variation in relative PFAS concentrations may indicate that Zhejiang and Shanghai could have different pollution sources. Furthermore, based on total PFAS concentrations measured and associated mass loading calculations, the authors estimated that ≥4000 kg of total PFAS is transported by rivers annually to the East China Sea.

PFOA and PFOS were the predominant pollutants found in China’s environmental media. Although the production and use of PFOA and PFOS have been significantly scaled down in many developed countries, they are still used in several industries in China; and contamination from industrial wastes is still considered a primary source of PFAS in the environment. Between 2003 and 2006, the annual production of PFAS-related chemicals increased significantly leading to the frequent detection of these compounds in the aquatic environment (Wang et al., 2015), and PFAS have been detected in seven major river systems and their main tributaries in China. For example, high concentration of PFAS was detected immediately beneath the industrial park in Fuxin, China. The groundwater immediately beneath the park had the highest PFOA concentration of 524 ng L^−1^. At other locations sampled for the study, the PFOA levels detected in drinking water from the public water supply system ranged from 1.3–2.7 ng L^−1^ (Bao et al., 2011).

The levels of PFAS have been monitored in major river systems in China, including the Pearl, Yangtze, and Haihe Rivers. In 2003, PFOA and PFOS were detected in the middle reaches of the Yangtze River at concentrations ranging from 0.2–297.5 and 0.1–37.8 ng L^−1^, respectively (Wang et al., 2015). Then, in 2004, PFOA and PFOS were detected in the lower reaches of the Pearl River at concentrations ranging from 0.85–13 ng L^−1^ and 0.9–99 ng L^−1^, respectively; and in the lower reaches of the Yangtze River at concentrations ranging from 2 to 260 ng L^−1^ and ND–14 ng L^−1^, respectively (So et al., 2007). The measured concentrations of PFOA and PFOS in the Pearl River were lower in 2013, with PFOA at 0.71–8.7 ng L^−1^ and PFOS at 0.52–11 ng L^−1^ (Liu et al., 2018). This reduction in PFOA and PFOS concentrations has been attributed to a decline in production and release from industrial sources due to the requirements of the Stockholm Convention (Wang et al., 2015).

The presence of PFAS in China’s major river systems is not surprising, as numerous industrial areas are positioned along these rivers. The Yangtze River, for example, flows through multiple urban, industrial, and commercial areas and is therefore vulnerable to PFAS pollution. Similarly, high concentrations of PFOA and PFOS have also been measured in the Haihe River, located in Northern China, especially in the areas near to the Bohai Economic Rim (Wang et al., 2015). The mean concentrations of PFAS detected across all of the tributaries to the Yellow River was 15.5 ng L^−1^ (Wang et al., 2016). In the Hanjiang River, the largest tributary to the Yangtze river which flows through Wuhan, China, PFOA and PFOS concentrations were measured at 8.9 and 568 ng L^−1^, respectively (B. Wang et al., 2013; X. Wang et al., 2013). The unregulated discharge of industrial wastewater is a major source of PFAS. For example, the Daling River, located in one of the most developed regions of northern China, had discharged 40.57 kg y^−1^ and 1.74 kg y^−1^ of PFOA and PFOS, respectively, into the Bohai sea (Li et al., 2017).

Jin et al. (2009) analyzed 13 surface water samples from remote areas of China, including Tianchi Lake, Benxi Shuidong, Xiaoqing Lake, Baotuquan, Dixiahualang, Jinbian Brook, Baofeng Lake, Huanglong Dong, Yuepu Lake, Kalakule Lake, and Tianshan Tianchi Lake. Twelve of the samples contained PFAS with maximum measured concentrations of 0.4 ng L^−1^ and 2.4 ng L^−1^ for PFOA and PFOS, respectively. The level of PFOS was higher in samples collected from urbanized areas than that of PFOA in the remote samples. The only samples in which PFOA and PFOS were not detected were collected from a buried river within a limestone cave in Liaoning province, known as Benxi Shuidong (Jin et al., 2009). Trace levels of PFOA and PFOS in these remote samples suggest that long-range transport is possible for PFAS.

In general, higher concentrations of PFAS were found in industrialized and urbanized areas than in remote, rural areas of China. This observation is consistent with established research which indicates that the increase in the concentration of PFAS in surface waters is related to trends in industrialization and urbanization, which are expected to increase across China (Chen et al., 2009; Bao et al., 2011; B. Wang et al., 2013; X. Wang et al., 2013; Lu et al., 2015; Li et al., 2017). Interestingly, the PFAS concentrations detected near industrialized or urban areas in China were similar to those found in surface waters in the USA, Canada, Japan, and Europe (Wang et al., 2015).

Few studies have investigated the presence of PFAS in the Indian environment. The presence of legacy PFAS in surface waters in India was first documented by (Yeung et al., 2009). They measured the levels of PFAS in Indian rivers, lakes, coastal seas, wastewater, and biota, with PFOA and PFOS being the most frequently detected. PFOS concentrations in the untreated wastewater were 12.0 ng L^−1^, and concentrations in water samples and aquatic biota ranged from 0.04–3.91 ng L^−1^ and 0.248–27.9 ng g^−1^, respectively. The highest concentration of PFOS in water samples (3.91 ng L^−1^) was recorded from the Cooum River, whereas that in animal tissue was detected in dolphins (*Platanista gangetica*) found in the Ganges River (27.9 ng g^−1^), indicating a bioaccumulation pathway. The concentrations of these pollutants in India were relatively low compared to those in other Asian countries such as China, South Korea, and Japan (Yeung et al., 2009).

An extensive study investigating PFAS pollution in groundwater and river water across the Ganges, India’s largest river, detected 15 PFAS in water samples (Sharma et al., 2016). This study represents a transect of approximately 2525 km, and includes samples collected from surface waters and groundwater (in the vicinity of the riverbank) across densely populated and industrialized areas and tributaries of the Ganges River. Surface water from the Ganges and the groundwater in this region are used as sources of drinking water. Among the detected PFAS, PFHxA (0.4–4.7 ng L^−1^) and perfluorobutanoic sulfonate (PFBS) (<Method Quantitation Limit (MQL) – 10.2 ng L^−1^) had the highest concentrations. The prevalence of these short-chain PFAS are due to the substitution of PFOA and PFOS during 2009–2016. The discharge of PFOA and PFOS to the Ganges River varied dramatically along the transect (0.2–190 and 0.03–150 g d^−1^, respectively), and in groundwater samples, perfluorobutanoic acid (PFBA) and PFBS had the highest concentrations among PFCAs and PFSAs, respectively (Sharma et al., 2016). The emissions from urban residents living along the bank of the Ganges River are likely responsible for PFCAs and PFSAs pollution, whereas PFAS pollution in groundwater is likely due to infiltration, spills, and the use of river water for irrigation purposes.

Nguyen et al. (2016) investigated the transport and fate of PFAS within an urban water reservoir in Singapore. The study focused primarily on comparing the relative concentrations of PFAS present in suspended sediments, water, and surficial sediments. PFOA was the most abundant PFAS detected in the aqueous phase, followed by PFOS and PFHxS, with average concentrations of 58, 31, and 25 ng L^−1^, respectively (Nguyen et al., 2016).

Duong et al. (2015) focused on the occurrence of PFAS in Vietnam and reported that PFOA and PFOS were among the most detected PFAS in urban river waters at maximum concentrations of 18 and 5.3 ng L^−1^, respectively; while the maximum concentrations of these pollutants detected in groundwater were 4.5 and 8.2 ng L^−1^, respectively. PFOA was detected in 98% of surface water samples and 36% of groundwater samples, while PFOS was detected in 59% of surface water samples and 45% of groundwater samples. Fourteen samples were also collected from the Red River, the second largest river in Vietnam. PFOA concentrations in the Red River were generally low, ranging from 0.16–0.52 ng L^−1^, and PFOS was detected at only one location at a concentration of 0.21 ng L^−1^ (Duong et al., 2015). Their results indicated that PFAS concentrations were subject to seasonal variation as concentrations were relatively higher in the rainy season versus the dry season suggesting that stormwater runoff could be a potential non-point source of PFAS pollution in Vietnam. Similarly, Lam et al. (2017) surveyed the occurrence of PFAS in surface, tap, and groundwater samples collected from eight different regions in Vietnam. PFOA and PFOS were consistently detected in surface waters, and their highest concentrations – 53.5 ng L^−1^ and 40.2 ng L^−1^, respectively – were detected in a sample from a river channel that directly received discharge of treated effluent from a WWTP. However, besides the aforementioned samples, the average PFOA and PFOS concentrations were primarily low in surface waters, measuring below 2.3 ng L^−1^ and 0.5 ng L^−1^, respectively. The average PFOA concentrations detected in tap water and groundwater – 0.14 ng L^−1^ and 1.93 ng L^−1^, respectively – were also relatively low at the Vietnam sampling sites. PFOS was not detected in tap water, and its average concentration in groundwater was low at 0.32 ng L^−1^. The highest PFAS concentrations in Vietnam were found in water samples collected from highly populated and industrialized areas; however, PFAS concentrations were generally lower in Vietnam compared to those reported from other industrialized countries.

Choi et al. (2017) surveyed the soils and waters near agricultural areas in South Korea and detected PFOA and PFOS in all samples, indicating the widespread contamination of PFOA/PFOS in waters used for agriculture in South Korea. They observed significant spatial variation in the concentrations of both pollutants, and their higher concentrations were caused by the WWTPs located upstream of the sample locations. Although PFAS were frequently detected, a large number of samples (92.3%) had PFOA and PFOS concentrations ≤50 ng L^−1^ (Choi et al., 2017).

Although PFOS has been regulated worldwide, numerous potential precursors that may eventually degrade into PFOS or PFOA are still being used. Ye et al. (2014) investigated the occurrence and distribution of PFAS precursors in the Tama River which flows into Tokyo Bay. They used a unique approach to convert all PFAS precursors into PFCA via chemical oxidation. The sum of PFCAs formed by oxidation was compared to the sum of initial PFAS present. They found high total concentrations of oxidized PFCAs in WWTP effluents; however, the ratio of oxidized PFCAs to pre-oxidation PFAS found in effluents were lower than those found in river water samples. This finding provides evidence for the decomposition of precursor compounds into PFAS during the wastewater treatment processes. Interestingly, higher ratios were also observed in upstream water samples; indicating that WWTPs were not the only source of precursor compounds.

In Australia, Gallen et al. (2017) screened the influent, effluent, and biosolid samples from 14 WWTPs for the presence of various PFAS. They reported concentrations of PFAS ranging from 0.98–440 ng L^−1^ in influent, 21–560 ng L^−1^ in effluent, and 5.2–150 ng g^−1^ in biosolids. Another recent study found PFAS in biosolids from 12 WWTPs across Australia (Sleep and Juhasz, 2020). The authors observed significant variation in the concentration of PFAS in biosolids across geographical locations, with concentrations ranging from 5.4–150 μg kg^−1^. Furthermore, 75% of biosolid samples contained PFOS, making it the most frequently detected contaminant with concentrations ranging from 4.7–86 μg kg^−1^.

Recycled wastewater used for irrigation purposes can serve as a diffuse source of PFAS and can affect groundwater quality. For example, at an agricultural site in Werribee South, near Melbourne, Australia, wastewater was used for irrigation and measurements from groundwater samples reported a mean PFOS concentration of 11 ng L^−1^ (maximum = 34 ng L^−1^), and a mean PFOA concentration of 2.2 ng L^−1^ (Szabo et al., 2018).

Investigations into PFAS pollution are ongoing at several military sites in Australia where AFFF was historically used in firefighting exercises. PFOA and PFOS attributed to AFFF use were detected at a military base in Williamtown, New South Wales, with maximum groundwater concentrations of 1800 ng L^−1^ and 5560 ng L^−1^, respectively. The concentrations of pollutants in the groundwater declined as the distance from source areas increased; however, PFOS concentrations were also detected off-base at concentrations above 200 ng L^−1^ (Goverment, 2015). Bräunig et al. (2017) also reported the occurrence of PFOS and PFOA near firefighting training areas in Oakey, a town in Queensland, Australia, with concentrations ranging from 170 to 14,000 ng L^−1^ and 50–600 ng L^−1^, respectively.

**Fig. 1.**
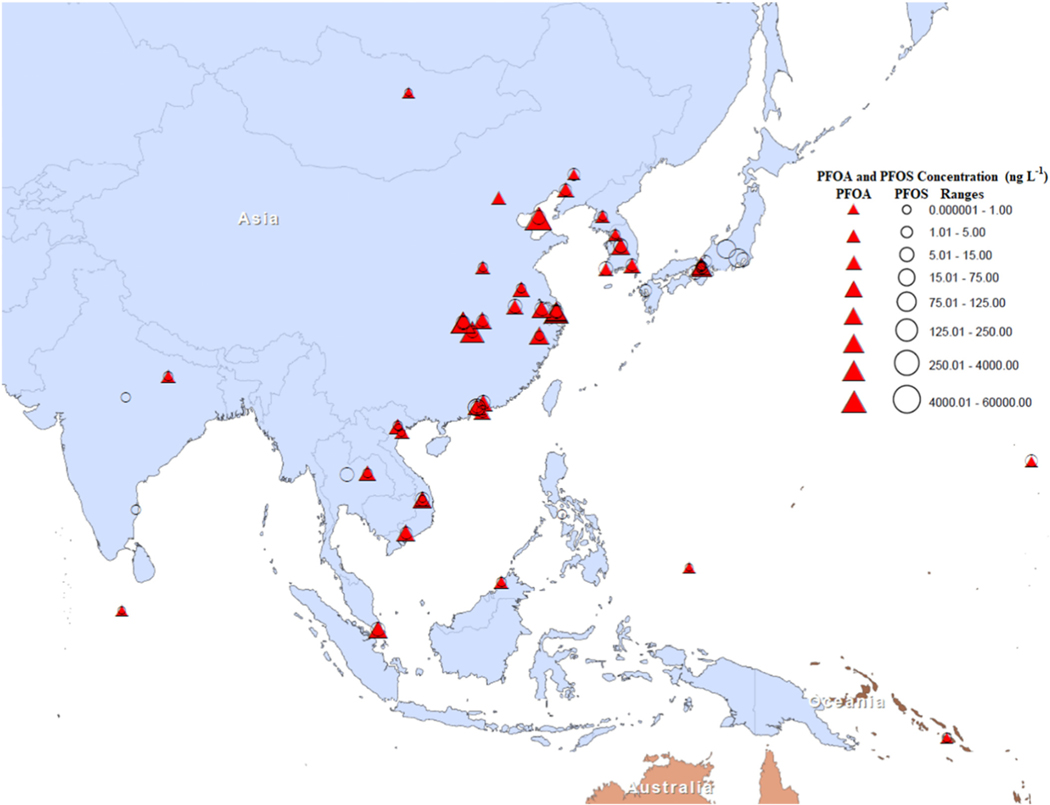
The spatial distribution of *per*- and polyfluorinated substances (PFAS) detected at various concentrations in the Asia-Pacific Region.

Snow and ice cores provide a useful record to analyze historical trends in environmental contamination from sources worldwide. Such methods are particularly relevant to PFAS, given their history of production, multiple source regions, and the varied mechanisms driving their global distribution, such as atmospheric transport B. Wang et al. (2013) and X. Wang et al. (2013). The authors studied the levels of PFAS in dated snow–ice cores collected from the high-altitude mountain glaciers on Mt. Muztagata and Mt. Zuoqiupo on the Tibetan Plateau. Ice cores from the Mt. Muztagata glacier in Western Tibet, representing the period of 1980–1999, had higher concentrations of PFOS (61.4–346 pg L^−1^) and PFOA (40.8–243 pg L^−1^). The ice cores from Mt. Zuoqiupo in Southern Tibet, representing the period 1996–2007, had a lower PFAS content compared to the core from Mt. Muztagata. PFOS concentrations in the cores from Mt. Zuoqiupo were lower (below the detection limit), while the measured PFOA concentrations ranged from 37.8–183 pg L^−1^ (B. Wang et al., 2013; X. Wang et al., 2013). Fig. 1 shows the spatial distribution of PFAS in the Asia-Pacific region.

The distribution and occurrence of PFOS and PFOA in different parts of the Asia-Pacific region indicate that water bodies are increasingly vulnerable to PFAS pollution. A summary of detection of PFOS and PFOA in the Asia-Pacific region in various environmental matrices is provided in Table S2. Countries that do not currently monitor PFAS levels in relevant water bodies should take note of these documented instances of occurrence of PFAS in this region. Although global agencies, such as the United Nations Environmental Programme (UNEP), have taken the initiative to monitor PFAS contamination in the developing countries of this region, few studies have been conducted due to a lack of resources, available technology, and skills.

### 5.2. Occurrence of PFAS in North America

PFAS have been documented in many different water sources, including rivers, lakes, groundwater, and the soil environment in North America. A study conducted by Boulanger et al. (2004) confirmed the presence of PFAS in the American Great Lakes, which represent nearly one-fifth of the continent’s readily accessible freshwater and the concentrations of PFOA and PFOS in Lake Ontario and Lake Erie ranged from 21 to 70 and 27–50 ng L^−1^, respectively. Similarly, a study by Silva et al. (2011) also confirmed the presence PFOA in Lake Superior, Lake Michigan, Lake Erie, Lake Huron, and Lake Ontario, with concentrations of the pollutant ranging from 0.75–1.2 ng L^−1^; 3.7–5.2 ng L^−1^; 3.4–7.2 ng L^−1^; 0.66–4.3 ng L^−1^; and 3.3–6.7 ng L^−1^, respectively. In the same study, PFOS concentrations measured in these lakes ranged from 0.14–0.40 ng L^−1^; 1.7–2.4 ng L^−1^; 2.5–3.4 ng L^−1^; 0.24–5.5 ng L^−1^; and 2.6–9.5 ng L^−1^, respectively. The temporal distribution of PFOS in Lake Ontario during the sampling events conducted in 2004, 2005, and 2010 showed minimal changes in mean concentrations of PFOS over that period (Furdui et al., 2008; Silva et al., 2011). The lowest concentrations of PFOS and PFOA were found in Lake Superior, and the highest were found in Lake Erie and Lake Ontario, which are both downstream from Lake Superior.

Surface water and groundwater are the primary sources of drinking water in North America, and PFAS are frequently detected in these water resources. A comprehensive study investigating the occurrence of 17 PFAS in source water and treated drinking water from 25 drinking water treatment plants across the United States (24 states) reported that PFAS were present in all drinking water samples with the cumulative PFAS average concentration ranging from less than 1 ng L^−1^ to 1102 ng L^−1^. The median concentration of PFAS in the source water (21.4 ng L^−1^) was higher than the median concentration in treated water (19.5 ng L^−1^). At least one of the 25 water treatment plants investigated in this study exceeded the US EPA LHA limit for drinking water (Boone et al., 2019). Another study found that three municipal water supply wells adjacent to Fairchild Air Force Base near Spokane, Washington, had concentrations of PFOA and PFOS above the US EPA LHA limit (AFB, 2017). Cui et al. (2020) found that PFAS concentrations in drinking water from Miami, Florida, exceeded the stipulated US EPA LHA limit with concentrations ranging from <70 ng L^−1^ to 2000 ng L^−1^ (Cui et al., 2020). These cases suggest that long-term monitoring and installation of water treatment technologies are required to mitigate the effects of water pollution.

In the USA, many state agencies perform drinking water source evaluation to monitor the levels of PFAS. For example, the Washington State Department of Ecology (WDOE) conducted a statewide survey of surface waters in Spring and Fall of 2008, which found PFOA and PFOS in concentrations ranging from below the limit of quantification (LOQ) to 95.6 ng L^−1^ and ND – 7.6 ng L^−1^, respectively. The highest concentrations were detected in West Medical Lake, which is likely related to wastewater discharged from the WWTP on the adjacent Medical Lake. The WDOE study noted that the concentrations detected in surface waters during the statewide survey are comparable to the average concentrations detected in similar surface water bodies in other states (Furl and Meredith, 2010; WDOE, 2010).

The New Hampshire Department of Environmental Services (NHDES) has also published results from ongoing drinking water contamination investigations. In June 2021, NHDES released a census stating that 78% of the 1880 sources of drinking water have been sampled and analyzed for the presence of PFAS. Overall, 30% of the wells were found to be contaminated with PFOA, PFOS, PFHxS, and PFNA. Furthermore, contaminant concentration levels in 103 wells exceeded the current NHDES MCL/Ambient Groundwater Quality Standard (AGQS). Note that NHDES has its own MCL/AGQS standards for PFAS which differ from the USA national standards. The MCL/AGQS values for PFOA, PFOS, PFHxS, and PFNA are 12 ng L^−1^, 15 ng L^−1^, 18 ng L^−1^, and 11 ng L^−1^, respectively; while the highest detected concentrations of these compounds during the above survey were 320 ng L^−1^, 2400 ng L^−1^, 73 ng L^−1^, and 830 ng L^−1^ (NHDES, 2021). It is important to note that while the contaminant levels in some of these water sources exceeded both the State and Federal advisory levels, some are currently not in use, some have treatment systems installed, and the management status of others is unknown.

In 2006, the New Jersey Department of Environmental Protection (NJDEP) detected low-level concentrations of PFOA and PFOS in 58% and 78% of water systems sampled, respectively. Thereafter, the NJDEP issued a health-based guidance level for PFOA at 400 ng L^−1^ and determined that additional monitoring was required. A second study was conducted between 2009 and 2010 and involved the analysis 10 PFAS analytes in 31 water systems using samples from untreated raw groundwater and surface water sources. Overall, PFAS were detected in 67% of samples, with PFOA being the most frequently detected in nearly 55% of samples. PFOA was detected in 92% of surface water samples with concentrations ranging from 6 to 100 ng L^−1^, and PFOS was detected in 33% of groundwater samples with concentrations ranging from 9 to 57 ng L^−1^ (NJDEP, 2014). In 2020, NJDEP revised its advisory limits for PFOA and PFOS in drinking water to 14 ng L^−1^ and 13 ng L^−1^, respectively. These values are among the lowest available health-based guidance levels for PFAS in drinking water throughout the United States.

In Southern California, the Orange County Water District (OCWD) reported detectable concentrations of PFOA and PFOS in water samples collected from five water agencies in Orange County, including Anaheim Public Utilities, the city of Fullerton, the city of Garden Grove, the City of Orange, and the Yorba Linda Water District. Excessive concentrations of (PFOS/PFOA > US EPA LHA limit) were detected in one of the wells in Anaheim and two of the wells in the City of Orange, and these wells were subsequently taken out of service (OCWD, 2016). In 2020, the California Division of Drinking Water issued drinking water advisory limits in the form of Notification Levels (NLs) and Response Levels (RLs) for PFOA and PFOS. The NL for PFOA and PFOS are 5.1 ng L^−1^ and 6.5 ng L^−1^, respectively, while the RL values for each pollutant are 10 ng L^−1^ and 40 ng L^−1^, respectively. If NLs are exceeded, the water agencies are required to report to the local elected officials and governing bodies regarding the detection of PFOS and PFOA in local water supplies, whereas the exceedance of RLs prevents the water agencies from serving water to the community (OCWD (Orange County Water District), 2021).

In Minnesota, Oliaei et al. (2013) examined PFAS contamination in areas adjacent to one of the top PFAS production facilities operated by 3M. Over five decades of production, this facility generated large quantities of PFAS and disposed of significant quantities throughout several sites. The 3M Cottage Grove facility is among the best-studied PFAS contamination and remediation sites globally; with the first investigations related to PFAS contamination having been initiated in 2002 by the Minnesota Pollution Control Agency. Since then five other sites associated with the 3M production facility were also investigated for PFAS pollution: Washington County landfill, the 3M Oakdale disposal site, the 3M Woodbury disposal site, the 3M Cottage Grove facility, and the Pine Bend landfill. The Washington County Landfill is a closed, unlined landfill site that historically accepted waste from the plant and registered very high concentrations of PFOA (42,000 ng L^−1^) and PFOS (2700 ng L^−1^) in groundwater. Oliaei et al. (2013) further reported that 3M WWTP sludge was disposed of at the partially lined Pine Bend Landfill site. As a result, significant levels of PFOA and PFOS pollution were measured in leachate, wastewater, and gas condensate from this site, as well as in the groundwater resources.

In 2004, elevated PFOA and PFOS concentrations were detected at the 3M Oakdale disposal site which was an unlined landfill used for industrial PFAS waste disposal between 1956 and 1960. Thereafter, in 2005, PFAS were detected in four municipal wells used to supply water to the Oakdale area. The historical events of PFAS pollution and subsequent remediation efforts at this facility indicate the gravity of the situation. The PFAS pollution in drinking water wells has been addressed by using alternative water supplies or the installation of activated carbon systems. In 2006, 3M agreed to install a $2.5 million activated carbon system to remove PFAS from the water supply in the City of Oakdale.

In addition to the pollution in municipal wells, PFAS has been detected in groundwater underlying the 3M facility. Elevated levels of PFOA and PFOS were detected in groundwater during Phase I remedial investigation, with PFOA concentrations ranging from 150,000–1,836,000 ng L^−1^ and PFOS concentrations ranging from 80,000–324,000 ng L^−1^; and the site is currently undergoing remediation using groundwater extraction and activated carbon treatment. High PFOA and PFOS concentrations were also measured in surface waters from the river cove, a small pond area that received wastewater effluent from the 3M plant. The maximum measured concentrations of PFOA and PFOS at this site were 18,200 ng L^−1^ and 3600 ng L^−1^, respectively.

In 2009, two locations in the Mississippi River, downstream from the 3M cove area, also registered varying concentrations of PFOA and PFOS, with average concentrations ranging from 17 to 94 ng L^−1^ and 15–90 ng L^−1^, respectively (Oliaei et al., 2013). A comprehensive study conducted during 2008 investigated the PFAS contamination in the Upper Mississippi River Basin and found that the majority of PFAS (around 80%) occurred at levels below 10 ng L^−1^; however, the maximum measured concentrations of PFOA and PFOS were 125 ng L^−1^ and 245 ng L^−1^, respectively, suggesting that other point sources were contributing to pollution in the river basin (Nakayama et al., 2010).

**Fig. 2.**
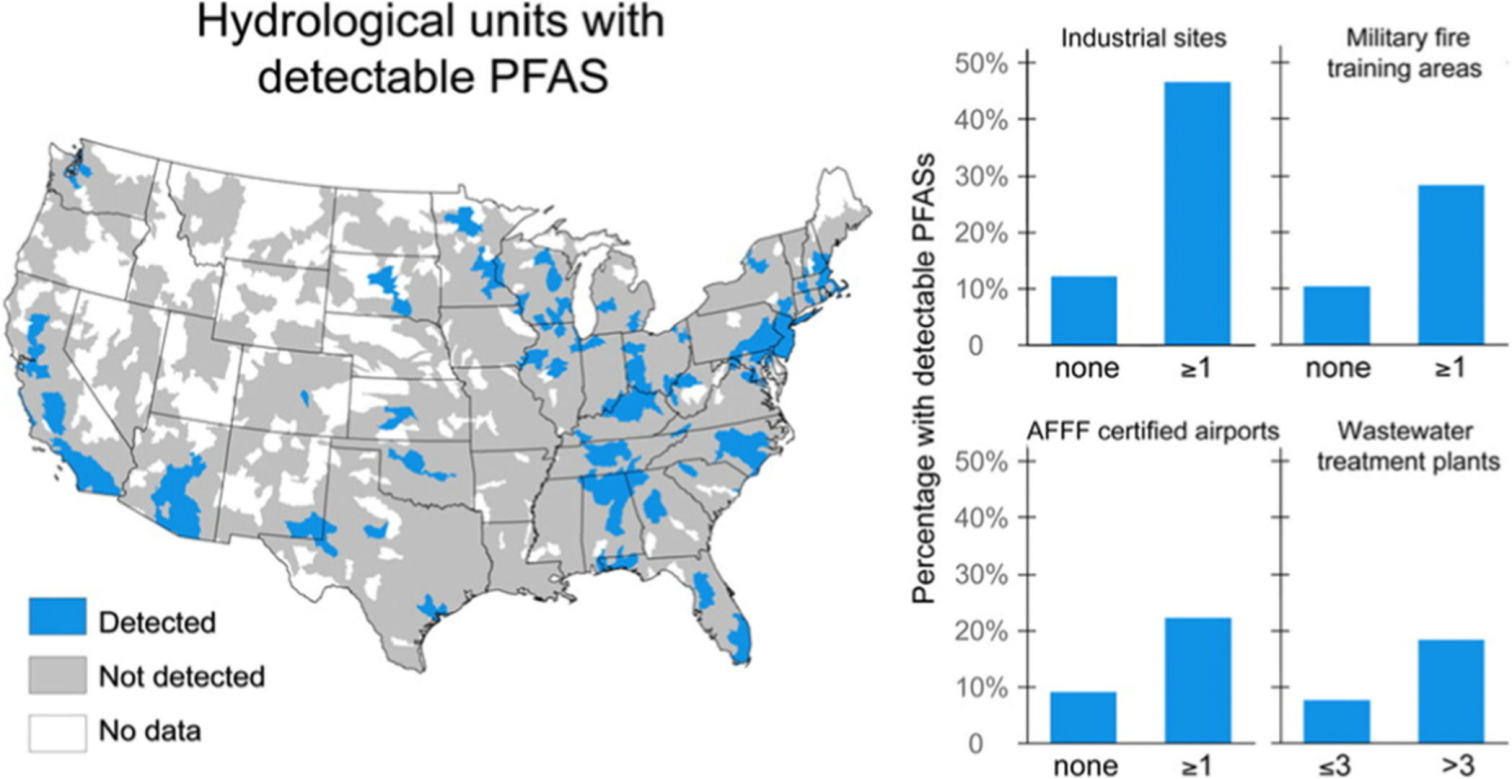
Detection of PFAS at industrial, military, airport, and municipal wastewater treatment plant sites across the United States. *Source:* Hu et al. (2016).

A 2017 spatial analysis of the data available from the US EPA UCMR 3 Program indicated a strong correlation between the PFAS concentrations in public water systems and their proximity to industrial sites where PFAS are used or manufactured, the number of nearby military fire training areas, and the number of wastewater treatment plants in the area. The study estimated that drinking water supplies for 6 million US residents exceeded the US EPA’s LHA for PFOA and PFOS. Furthermore, the study also indicated some critical data gaps, including unavailable data for small water systems and private wells, from which nearly one-third of the American public receives its water supply (Hu et al., 2016). Fig. 2 shows the occurrence and distribution of PFAS at various locations across the USA, including areas where no data are available.

Plumlee et al. (2008) evaluated PFAS concentrations in recycled water from five WWTPs in California. Four of these plants employed tertiary treatment methods, while the fifth treats primary effluent in a constructed wetland. Both PFOA and PFOS were detected in recycled water at concentrations ranging from 10 to 190 ng L^−1^ and 20–190 ng L^−1^, respectively. Effluent contaminant concentrations were compared to those of surface and groundwater from the Upper Silver and Coyote Creeks in San Jose, where recycled water was evaluated as a potential means to augment flow. Although wastewater was not being discharged, the stream samples collected from the Upper Silver and Coyote Creeks nevertheless contained PFOA and PFOS at concentrations of 8–36 ng L^−1^ and 5–56 ng L^−1^, respectively. The sources of PFAS in surface waters were attributed to urban runoff and atmospheric deposition. Groundwater samples that were taken from near the creeks also contained PFOA and PFOS at concentrations ranging from ND – 18 ng L^−1^ and 19–87 ng L^−1^, respectively. The presence of PFAS in groundwater was likely due to infiltration from the stream; however, other sources could not be ruled out (Plumlee et al., 2008).

**Fig. 3.**
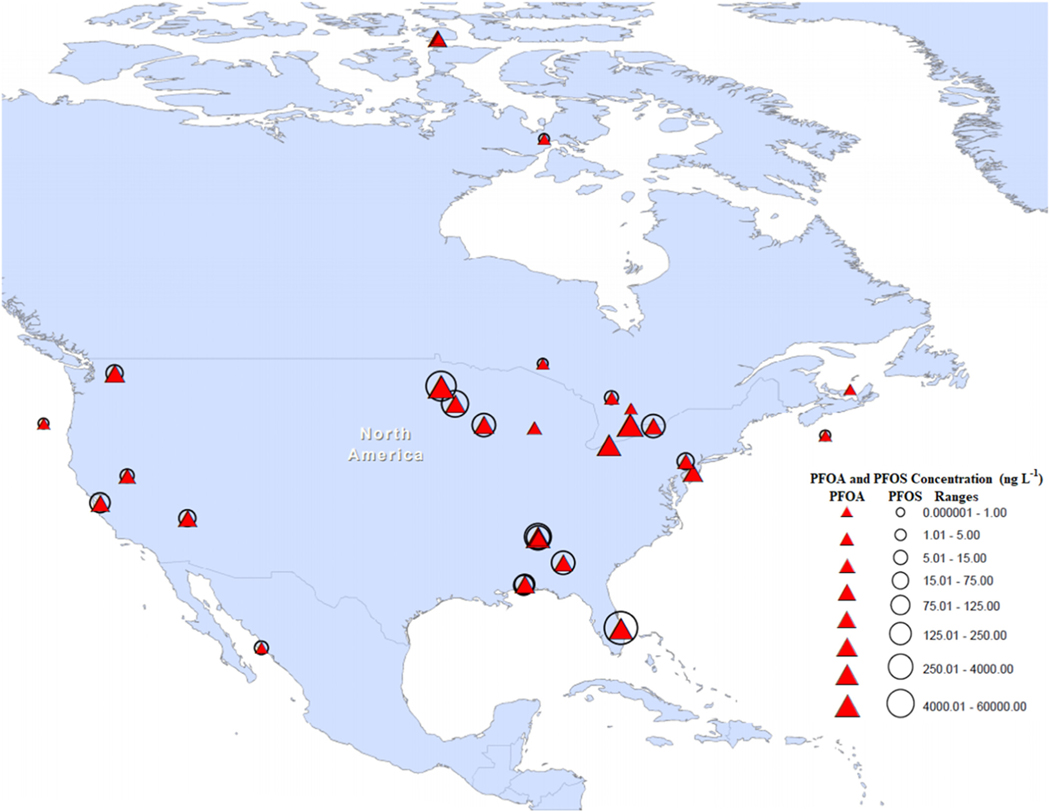
Occurrence and distribution of PFAS detected at various concentrations ranges in the North American Region.

Extensive PFAS pollution due to wastewater discharges has also been reported in the Las Vegas (Las Vegas Wash and Lake Mead) and Reno Watersheds (Truckee River, Lake Tahoe, and Pyramid Lake), the two major urban watersheds in the Western United States. The total PFAS concentration in surface waters of the Las Vegas Wash and Truckee River were 2234 ng L^−1^ and 441.7 ng L^−1^; while in sediments it was 345.7 μg kg^−1^ (dry weight) and 272.9 μg kg^−1^, respectively. Lake Mead, which is located downstream from the Las Vegas Wash, is a source of drinking water to 30 million residents and has registered seasonal PFAS concentrations of 271.9 and 14.8 ng L^−1^ in winter and summer, respectively (Bai and Son, 2021). Fig. 3 shows the widespread distribution and occurrence of PFAS in North America.

In a study published in 2017, surface water collected from rural, urban, and AFFF-impacted sites in Canada was analyzed using sensitive liquid chromatography-tandem mass spectrometry (LC-MS/MS) and 23 different PFAS were detected, of which perfluorohexane sulfonamide (FHxSA) was most prevalent. The concentrations of FHxSA in all urban and AFFF-impacted sites ranged from 0.04–19 ng L^−1^, indicating the widespread presence of perfluorohexanesulfonate (PFHxS) precursors in the Canadian waters (D’Agostino and Mabury, 2017). Benskin et al. (2012) reported perfluoroalkyl acids in the Canadian Atlantic and Arctic Oceans. They studied the spatial distribution of C4, C6, and C8 perfluoroalkyl sulfonates, C6–C14 PFCAs, and perfluorooctane sulfonamide in the Atlantic and Arctic Oceans, including the previously unstudied coastal waters of North and South America and the Canadian Arctic Archipelago (Benskin et al., 2012).

In their study, Chu and Letcher (2017) analyzed sediment samples collected from various locations in the Great Lakes’ basins and detected the presence of side-chain fluorinated polymer surfactants which are typically used as primary components of fabric protector sprays, furniture, and textiles (Chu and Letcher, 2017). They analyzed 15 sediment samples from western Lake Erie and Saginaw Bay (Lake Huron) using tandem mass spectroscopy (MS/MS) and quadrupole time-of-flight mass spectrometry (Q-TOF-MS). The results indicated that perfluorooctane sulfonamides (PFOSAs) were present in all sediment samples, with concentrations ranging from 0.18–461.59 ng g^−1^ (dry weight). In contrast, perfluorobutane sulfonamides were detected in 80% of sediment samples, with concentrations ranging from <0.03 to 24.08 ng g^−1^. They also analyzed 13 soil samples collected from agricultural areas in Southern Ontario, Canada, amended with wastewater biosolids, and detected PFOSAs in all soil samples with concentrations ranging from 41.87–622.46 ng g^−1^ (dry weight) with a mean concentration of 236.36 ng g^−1^. A summary of detection of PFOS and PFOA in North American region in various environmental matrices is provided in Table S3.

### 5.3. Occurrence of PFAS in South America and the Caribbean

There is growing concern regarding the occurrence of PFAS in environmental media in South America and the Caribbean region. Recently, many countries in this region have begun actively monitoring the presence of these pollutants in the environment. One of the earliest studies, conducted by Quinete et al. (2009), measured PFAS concentrations in drinking water from various districts in Rio de Janeiro and surface water samples from the Paraiba do Sul River, one of the largest rivers in southeastern Brazil. The Paraiba do Sul River flows through Brazil’s most important urban and industrial centers, including Rio de Janeiro and Sao Paulo. PFOA and PFOS were both found in all drinking water samples collected during this study with concentrations ranging from 0.35–2.82 ng L^−1^ and 0.58–6.70 ng L^−1^, respectively. Furthermore, both pollutants were detected in river water samples at concentrations ranging from ND – 1.22 ng L^−1^ and ND – 1.32 ng L^−1^, respectively. In another study, 16 different PFAS were detected in tap water and bottled water samples collected from the Porto Alegre metropolitan area. PFOS was detected in all tap water samples, whereas PFOA was detected in 33% of samples; both pollutants occurred at mean concentrations of 16 ng L^−1^. However, PFOS was not detected in bottled water, whereas PFOA was detected in 33% of samples at a mean concentration of 7.6 ng L^−1^ (Schwanz et al., 2016). Given that both bottled water and tap water are contaminated with some level of PFAS, drinking water certainly provides an exposure pathway for the population of Brazil.

Munoz et al. (2017) investigated the occurrence and spatial distribution of 22 PFAS in groundwater, surface water, and sediments in tropical areas, including French Guiana, Guadeloupe, Martinique, Mayotte, and Reunion. This research was the first to confirm that PFAS were present in these regions; however, concentrations were significantly lower than those detected in metropolitan France. PFAS were detected in groundwater close to several industrial sites, including oil refineries and electrical power plants, but the highest concentration of PFOS was detected in sediment and surface water samples. Most PFAS contamination was attributed to the firefighting training activities which took place near those sites (KEMI, 2015; Munoz et al., 2017).

PFAS are still used for specialty production purposes in several countries within South America and the Caribbean. In Brazil and Colombia, the large-scale production and use of PFAS-based pesticides such as sulfluramid in local agriculture is an important source of PFAS contamination. Sulfluramid contains Ethyl Perfluorooctane sulfonamide (EtFOSA); a PFOS-precursor which is used in the production of insect baits with sulfluramid for controlling leaf cutter ants (Rauert et al., 2018; UNEP, 2020). It is important to note that the production of sulfluramid in Brazil doubled from 30 to 60 tons per year from 2003 to 2013, with significant exports made to nearby countries. Furthermore, it is estimated that Brazilian sulfluramid production between 2004 and 2015 could potentially have contributed to the release of 167–487 tons of PFOS/FOSA into the environment (Gilljam et al., 2015). Their model simulations predict that once EtFOSA is released into the environment, it preferentially adsorbs to sediments. The transformation products of EtFOSA – perfluorooctane sulfonamide (FOSA) and PFOS – are highly mobile and immediately impact the surface and groundwater. Evidence of such transformation is documented from surface water contamination by FOSA and PFOS in Brazil and Columbia with uniquely high FOSA/PFOS ratios. The origin of these pollutants was attributed to the widespread use of sulfluramid insecticides in this region (Gilljam et al., 2015; Rauert et al., 2018). A summary of detection of PFOS and PFOA in the South America and the Caribbean region in various environmental matrices is provided in Table S4.

**Fig. 4.**
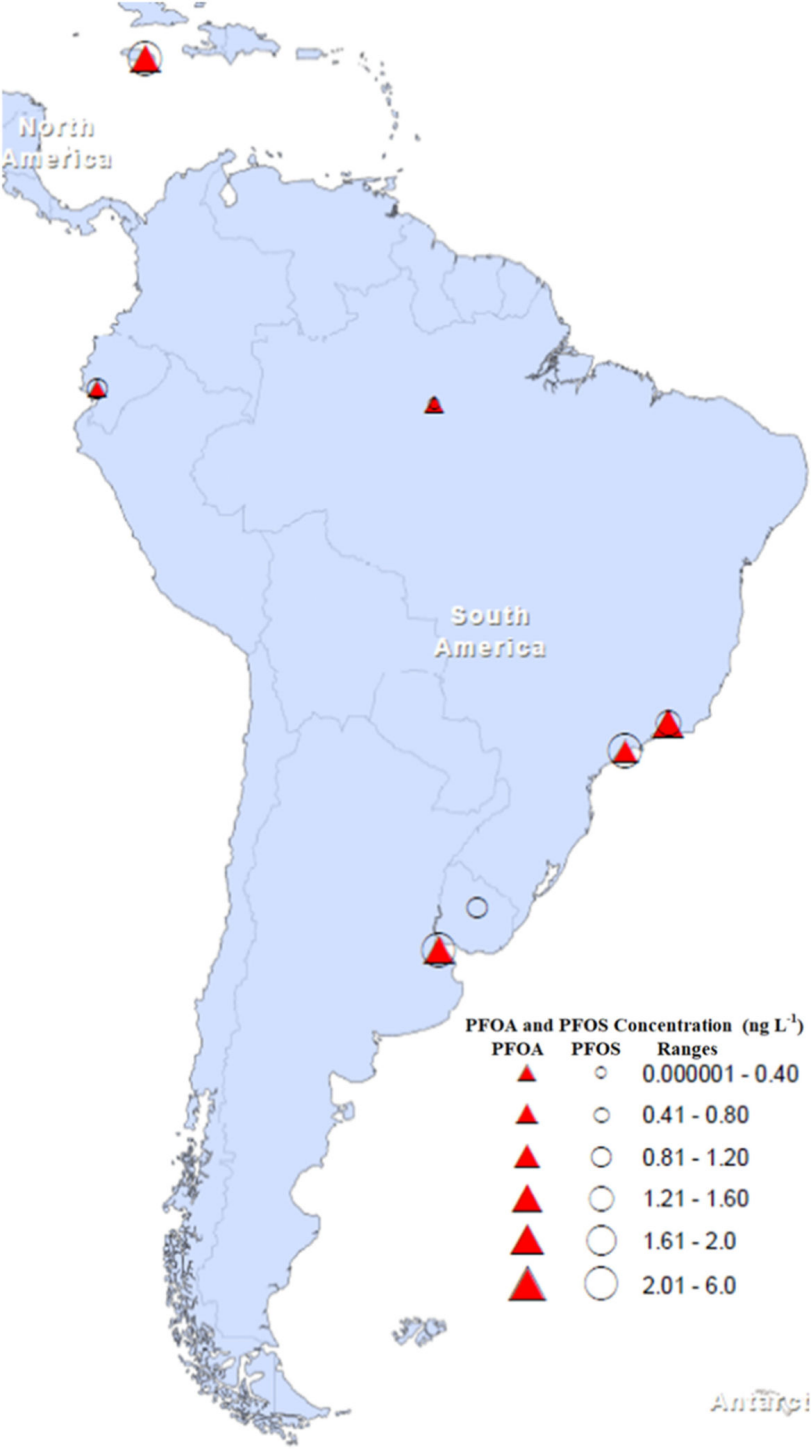
The occurrence and distribution of PFAS detected at various concentrations in surface waters across the South American and Caribbean region.

Despite measurable levels of PFAS being detected in the atmosphere at various locations throughout the 33 countries which make up South America and the Caribbean, there have been no concerted efforts to monitor the levels of these pollutants in the environment. The second global monitoring report for POPs under the Stockholm Convention (2017) acknowledged a shortage of monitoring data on PFAS within water systems in Latin America and the Caribbean. Currently, Uruguay is the only country that maintains a national program to monitor PFOS. Since these monitoring activities were recently initiated, no significant data are currently available (Fig. 4).

### 5.4. Occurrence of PFAS in the European region

The widespread occurrence of PFAS in various environmental matrices (surface water, groundwater, soil, and in aquatic biota) has been documented in many European countries. A phase-wise, long-term study monitoring the levels of PFAS in surface waters was conducted between 1999 and 2018 in Hesse, Germany. This study investigated the temporal variation and overall trends in PFAS occurrence and reported that detection frequency and maximum concentrations of PFOA decreased during the study period, whereas the detection frequency of short-chained PFAS increased especially after 2014 (Janousek et al., 2019). Another study showed that PFOA and PFOS concentrations in the Rhine River ranged from ND – 9 ng L^−1^ and 26 ng L^−1^, respectively (Skutlarek et al., 2006; Exner and Färber, 2006). The Ruhr, Emscher, and Lippe rivers – main tributaries of the Rhine River – had concentrations of PFOA and PFOS ranging from 21 to 48 ng L^−1^ and 5–18 ng L^−1^, respectively. The Rhine-Herne and Wesel-Datteln Canals contained PFOA at concentrations of 34 and 36 ng L^−1^ respectively. Lake Moehne also contained PFOA and PFOS with concentrations of 654 ng L^−1^ and 17 ng L^−1^, respectively (Skutlarek et al., 2006; Exner and Färber, 2006).

A few studies have investigated the vulnerability of European river systems to PFAS pollution. The Mediterranean rivers such as Ebro and, Guadalquivir rivers, in particular, are very vulnerable to PFAS pollution as they flow through densely populated urban areas. Extensive PFAS contamination has been reported in surface water, sediments, and aquatic biota from the Ebro and Guadalquivir River basins in Spain (Lorenzo et al., 2016). The authors documented a total of 21 PFAS from these river basins, PFBA, perfluoropentanoic acid (PFPeA) and PFOA were the most frequently detected. The maximum measured concentration of PFBA was 251.3 ng L^−1^ and 742.9 ng L^−1^ in water samples from the Ebro and Guadalquivir River basins, respectively. In the sediments from the Ebro River, PFOA was found in higher concentrations (maximum = 32.3 ng g^−1^), whereas PFBA was found in higher concentrations in sediment samples from the Guadalquivir River (maximum = 63.8 ng g^−1^). Bioaccumulation of PFAS differed significantly in biota from these basins. Twelve different PFAS were detected in fish tissue samples from the Ebro River, whereas only PFOS was detected in samples from the Guadalquivir River. The significantly different concentrations of PFAS in water, sediment, and aquatic biota from these two basins suggest that there are different pollution sources for either basin (Lorenzo et al., 2016).

In the Llobregat River ecosystem in northeastern Spain, PFAS concentrations in water, sediments, and aquatic biota were measured at 0.01–233 ng L^−1^, 0.01–3.67 ng g^−1^, and 0.79–431 μg kg^−1^, respectively (Campo et al., 2015). The higher concentrations of PFAS in aquatic biota samples suggest that bioaccumulation pathways in the Llobregat River are similar to those observed in Jucar River (Campo et al., 2016, 2015). Between 2010 and 2012, a large-scale study spanning 32 cities in Germany and Spain analyzed samples of ultrapure water, tap water, and treated wastewater. There were 148 water samples analyzed for the presence of 21 PFAS, and 88% contained at least one quantifiable amount of PFAS. Overall, 54% of all tap water samples were contaminated with PFBA at concentrations ranging from 2.4–27 ng L^−1^ (Llorca et al., 2012). Similar occurrence studies across 14 major European rivers, including the Rhine, Danube, Elbe, Oder, Seine, Loire, and Po, showed that river systems are becoming increasingly polluted with PFAS. Overall, the highest concentration of any PFAS was 200 ng L^−1^, which was measured for PFOA in the Po River; and the total estimated discharge of PFOA into European rivers was 14 Mg y^−1^ (McLachlan et al., 2007).

Analysis of river water samples collected downstream of a fluorochemical production plant in Dordrecht, Netherlands, indicated the presence of the novel PFAS, GenX chemicals, with the highest concentration (812 ng L^−1^) measured near the plant. The authors also analyzed the drinking water samples from adjacent municipalities and found that three of the four local municipalities had GenX chemicals in their drinking water at concentrations of up to 11 ng L^−1^ (Gebbink et al., 2017). This is yet another study that demonstrates that areas proximal to PFAS production facilities or water treatment plants are highly vulnerable to pollution by these compounds.

The use of biosolids from wastewater treatments plants in agriculture is a known source of PFAS pollution in river systems, which suffer due to the surface runoff originating from agricultural lands. Studies have shown that extensive land-based application of wastewater biosolids for agricultural activities in the Brilon–Scharfenberg area has substantially polluted a large portion of the upper Moehne River in Heidelberg. In this region, the concentrations of PFOA and PFOS in river water were measured at 3640 and 193 ng L^−1^, respectively (Exner and Färber, 2006). Residential areas, such as Neheim, which use the Moehne River water as a drinking water source also reported a high PFOA concentration of 519 ng L^−1^ in drinking water samples. Similarly, samples collected from other areas, such as Ruhr, showed the presence of PFOA at concentrations higher than the recommended US EPA Health Advisory level.

Sometimes PFAS have been detected in areas far away from urban and industrial zones; indicating that the PFAS contamination in surface waters is extensive and suggesting that long-range atmospheric transport is a potential concern. For example, a recent study in the Maltese Islands, Central Mediterranean, found that PFOA and PFOS were present in surface water samples with concentrations ranging from ND – 16 ng L^−1^, and <LOD to 8.6 ng L^−1^, respectively. Furthermore, PFOA was detected in 95% of the surface water samples. Interestingly, the authors noted that detection of PFAS in this remote island is attributed to precipitation, and the detected PFAS concentrations measured in precipitation ranged from 0.38–6 ng L^−1^. These findings suggest that long-range atmospheric transport of PFAS can threaten remote aquatic environments through precipitation (Sammut et al., 2017).

**Fig. 5.**
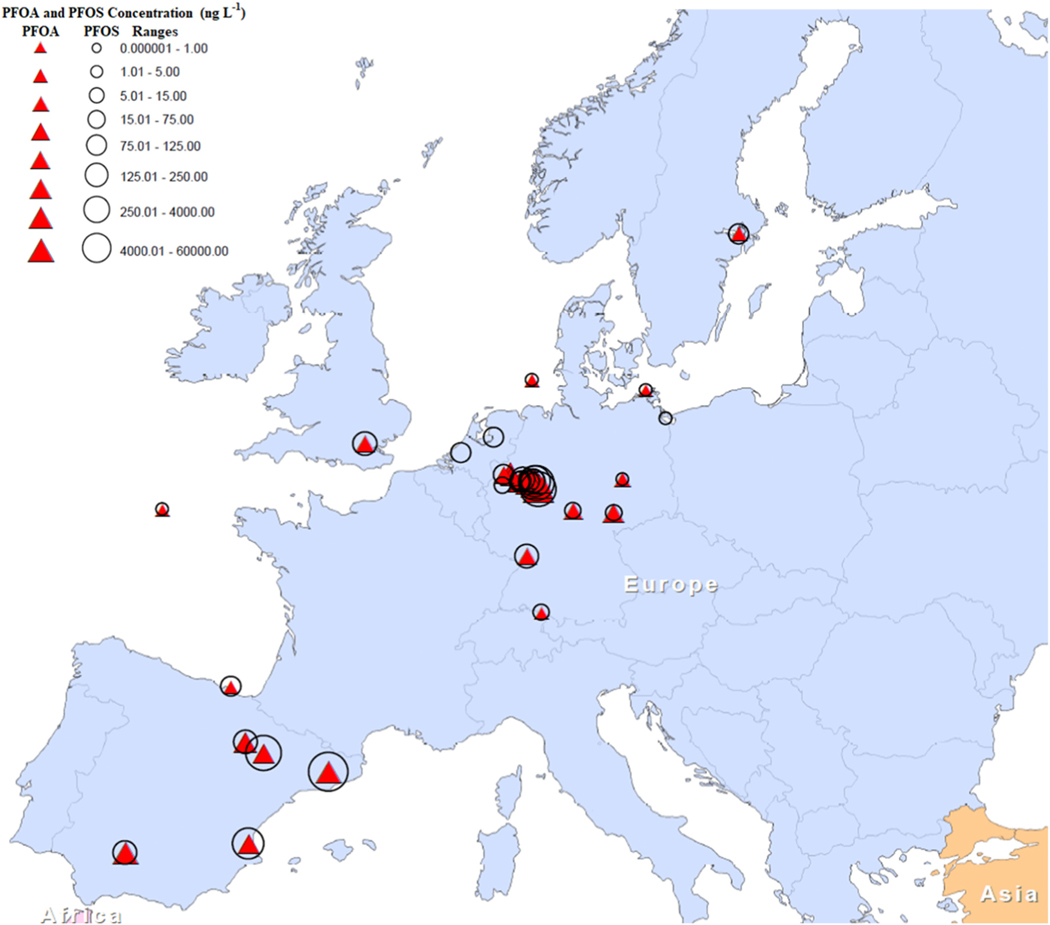
Occurrence and distribution of PFAS detected at various concentrations in water resources across the European region.

In 2014, the Swedish Water and Wastewater Association conducted a nationwide survey of drinking water, surface water, and groundwater quality. The results of this survey indicated that 22% of all water samples contained PFAS above set detection limits. PFOA and PFOS were the most frequently detected PFAS, and they were found more regularly in surface water samples than in groundwater samples. In raw drinking water samples, the maximum concentrations of PFOA and PFOS were 130 ng L^−1^ and 400 ng L^−1^, respectively (Banzhaf et al., 2016). A summary of detection of PFOS and PFOA in the European region in various environmental matrices is provided in Table S5. Fig. 5 shows the extent of PFAS contamination in the European region.

### 5.5. The occurrence of PFAS in the African region

Very few studies have examined the occurrence and distribution of PFAS across the African continent. Groffen et al., 2018 conducted a comprehensive reconnaissance study that examined the occurrence and distribution of 15 PFAS in river water, sediments, and aquatic biota from the Vaal River at three sites, Fischgat, Vaal Barrage, and Thabela Thabeng, which represented industrial, mining, and agricultural zones, respectively. PFAS were detected in all environmental media, and PFOS was the most frequently detected pollutant in aquatic biota samples, whereas PFPeA was predominantly detected in river water samples. The average concentrations of PFAS ranged from <LOQ to 38.5 ng L^−1^ in water; <LOQ in sediment; and <LOQ to 34.0 ng g^−1^ in aquatic biota (Groffen et al., 2018).

The UNEP and Global Environmental Fund have conducted research on the occurrence of PFAS in Mali and Kenya and have confirmed the presence of PFAS in surface waters. The surface water samples collected from the Niger River (Mali) and Sabaki River estuary (Kenya) contained PFOS at concentrations of 4.7 ng L^−1^ and 4.6 ng L^−1^, respectively (UNEP, 2017). Ahrens and Bundschuh (2014) found the presence of PFOA and PFOS in surface water collected from Lake Tana in Ethiopia. PFOA was detected in 60% of the surface water samples, with concentrations ranging from ND – 0.69 ng L^−1^; and PFOS was detected in 40% of the samples with concentrations ranging from ND – 0.22 ng L^−1^ (Ahrens and Bundschuh, 2014). During the MONitoring NETwork (MONET) pilot study in Africa, data were collected from passive samplers deployed during 2013–2014 in the surface waters of Congo, Egypt, Kenya, Mauritius, Morocco, and Nigeria. The Nigerian site had the highest PFOS concentrations of 1.39 ng L^−1^. The lowest concentrations of PFOS were detected in Morocco and the Congo at concentrations of 0.035 ng L^−1^ (UNEP, 2015).

**Fig. 6.**
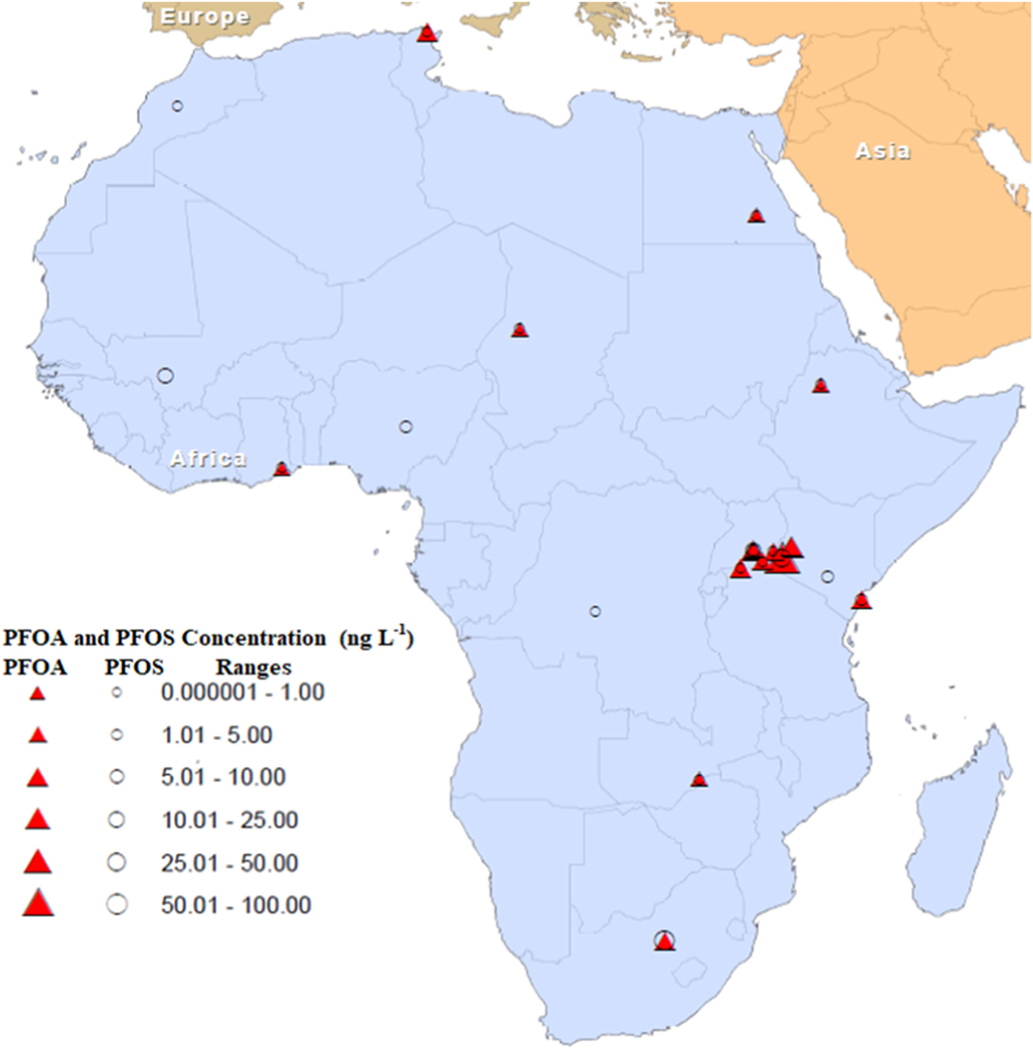
Occurrence and distribution of PFAS detected at various concentrations in surface water and groundwater in the African region.

Another study found seven PFCAs and three PFSAs in wastewater sludge samples, which were collected from various industrial and municipal areas in Nigeria. The concentrations of PFCAs and PFSAs ranged from 0.010–0.597 and 0.014–0.540 ng g^−1^, respectively, and even wastewater from hospitals’ treatment plants contained measurable levels of PFAS (Sindiku et al., 2013). A summary of detection of PFOS and PFOA in the African region in various environmental matrices is provided in Table S6. Although the detected pollutant concentrations were lower than those reported from other parts of the world, the extent of PFAS monitoring in Nigeria–and Africa as a whole–is minimal (Fig. 6). Further research is required to understand the distribution of other PFAS in wastewater and the environment.

## 6. Novel PFAS species

PFOS and PFOA were phased out of use in the early 2000s. Since then, various alternative/novel PFAS have been introduced. These novel PFAS include: perfluoroalkyl ether sulfonic acid (PFESA); perfluoroalkyl ether carboxylic acids (PFECAs); PFBS; PFBA; perfluoropolyether (PFPEs); polyfluoroalkyl phosphate diesters (diPAPs); 6:2 fluorotelomer sulfonate (FTSA); perfluorinated sulfonamidoacetic acids (FOSAA); 6:2 chlorinated polyfluorinated ether sulfonate (F53B); and GenX PFAS which include hexafluoropropylene oxide (HFPO) dimer acid and its ammonium salt (DuPontTM, 2010; Buck et al., 2011; B. Wang et al., 2013; X. Wang et al., 2013; Ahrens and Bundschuh, 2014; Newton et al., 2017; Pan et al., 2018; USEPA, 2018; Chen et al., 2019). These novel PFAS have gained acceptance in various industries as usable alternatives.

The PFOS used in the electroplating industry and firefighting foam manufacturing is being replaced with PFESA, FTSA, PFBS, and F53B; and the PFOA used in the manufacturing of high-performance fluoropolymer materials is being replaced by PFECA and GenX chemicals (DuPontTM, 2010; Buck et al., 2011; B. Wang et al., 2013; X. Wang et al., 2013; Ahrens and Bundschuh, 2014; Newton et al., 2017; USEPA, 2018; Pan et al., 2018; Chen et al., 2019; Wang et al., 2017). These novel PFAS have a different chemical structure in which fluorine atoms have been replaced by chlorine and hydrogen, and oxygen atoms have been inserted into their perfluorinated chains. Due to these significant structural differences, these alternatives were expected to be less recalcitrant compared to PFOS and PFOA; and could, therefore, easily breakdown into less toxic byproducts (DuPontTM, 2010; Buck et al., 2011; Wang et al., 2017). However, results from studies that have documented the persistence and occurrence of these novel PFAS in the environment have shown that this may not be the case. For example, both PFBS and GenX chemicals are toxic, persistent, and mobile in surface and subsurface environments (USEPA, 2018). A final version of the human health toxicity assessment released in 2021 by the EPA has proposed chronic and sub-chronic reference dose values – a daily dose ingested by an individual over a lifetime that may not cause adverse health impacts – for these chemicals. The chronic reference dose of PFBS was set at 0.0003 (3×10^−4^) mg kg^−1^ d^−1^, whereas for the GenX chemicals it was set at 0.000003 (3×10^−6^) mg kg^−1^ d^−1^, and sub-chronic dose at 0.00003 (3×10^−5^) mg kg^−1^ d^−1^, respectively (USEPA, 2021). Due to their persistent nature, as well as their bioaccumulative tendencies and toxic properties, GenX chemicals were added to the Candidate List of Substances of Very High Concern in the Netherlands in 2019 (Gebbink and van Leeuwen, 2020).

The novel PFAS are primarily released into the environment via industrial sources, such as waste streams generated during manufacturing processes. Recently, the release and occurrence of alternative PFAS, including short-chain PFAS and their precursors, has become a cause of concern. Since 2009, 455 newly identified PFAS, including nine perfluorinated and 446 polyfluorinated compounds, have been identified (Xiao, 2017). Many of these identified compounds could be potential precursor compounds to PFOS and PFOA. The release of PFAS from conventional WWTP effluent is well-documented; however, it is essential to identify unknown precursor compounds to estimate PFOS and PFOA secondary formation from WWTP effluent and other indirect sources (Xiao, 2017). Therefore, communities that were exposed to traditional PFAS have already been exposed to novel PFAS.

In the USA, several novel PFAS were recently detected in the Tennessee River downstream of fluorochemical manufacturing facilities near Decatur, Alabama. The detected pollutants included polyfluorinated carboxylic acids, polyfluorinated sulfate, and a series of perfluorobutane sulfonamido substances (Newton et al., 2017). In Minnesota, four sites used by the 3M facility were also found to be contaminated by legacy and novel PFAS, including PFBS, PFBA, PFPeA, and PFHxS (Scher et al., 2018).

Similarly, Gebbink and van Leeuwen (2020) documented the widespread occurrence of GenX chemicals in water samples collected near a fluorochemical processing facility in the Netherlands. The presence of GenX chemicals was reported in the waste stream (mean = 2.2 μg L^−1^) originating from the facility; effluent from the onsite WWTP (mean = 134 μg L^−1^); effluent from the municipal WWTP that receives pre-treated wastewater from the facility (mean = 375 μg L^−1^); and in surface water sampled upstream from the facility (mean = 0.75 ng L^−1^) (Gebbink and van Leeuwen, 2020).

The occurrence of novel PFAS such as PFBS, PFECA, and PFESA has also been reported in surface water and groundwater in China, the USA, the UK, Sweden, Germany, the Netherlands, Australia, and Korea (Bräunig et al., 2017; Pan et al., 2018; Marchiandi et al., 2021). There is significant variation in the worldwide distribution of novel PFAS, especially in terms of detection frequency, pollution concentrations, and location of occurrence. This is mainly because legacy PFAS were phased out long ago in North America and Europe, whereas the developing regions of Asia, Africa, and Latin America are still using legacy PFAS to some extent (Pan et al., 2018).

## 7. Conclusions and future perspectives

The occurrence and distribution of PFAS, including novel species, indicates that these compounds are frequently detected in the environment despite having been phased out. A review of multiple worldwide regions indicates that PFAS are distributed throughout the environment, irrespective of the level of economic/industrial development. This suggests that household items and consumer products could be the potential sources of PFAS in developing countries. PFAS have also been detected in environmental media located far away from industrial point sources, such as the aquatic resources of developing/non-industrial countries, remote areas such as the Tibetan mountains, and the vast water bodies such as the Pacific and Atlantic oceans. These results confirm that global long-range atmospheric transport is a pathway of PFAS distribution.

The widespread occurrence of these substances and their potential impact on human health remain primary public concern as recent epidemiological studies conclusively demonstrated the adverse health effects of PFAS exposure. Recently, regulatory agencies have recommended a series of screening/advisory values for drinking water have been recommended. In the US, additional regulatory measures are in various stages of being promulgated as more evidence becomes available. Currently, the US EPA has included PFAS on the contaminant candidate list (CCL5) which means that their levels are monitored but are not subject to US EPA’s drinking water regulation. The UNEP’s Stockholm Convention has included many PFAS on the persistent organic pollutant list. It has also restricted the use of firefighting foam containing PFOS, and its salts, if all the releases arising from such activities are not contained. These efforts demonstrate a proactive approach and speak to the environmental stewardship and relationship between stakeholders and regulatory agencies as they seek to protect human health and the environment.

In this review, we primarily focused on PFOA and PFOS. Future research should focus on potential PFAS precursors and their tendency to degrade/transform into PFAS such as PFCA, PFOS, and PFOA, under biotic and abiotic degradation processes. As such, the accurate determination and quantitation of short-chained PFAS and PFAS telomers formed during the degradation processes remains a critical area of research. At this time, identifying all precursors of PFAS remains a significant challenge and such research is needed to accurately quantify the mass influx of PFOS and PFOA entering the environment. Although major manufacturers have phased out legacy PFAS, the proposed novel/alternative PFAS–initially thought to be environmentally benign–may in fact be just as problematic as their predecessors. Many aspects of novel PFAS remain to be investigated including their analytical quantitation, bioaccumulative potential in aquatic biota, fate and transport in the surface and subsurface environment, as well as the toxicity of novel PFAS and their metabolites. Our review provides important baseline information on the occurrence and distribution of PFAS at a continental scale, as well as the current requirements for their management and mitigation of PFAS-associated pollution.

## Supplementary Material

Supplementary Material

## Figures and Tables

**Table 1:** Concentration limits for PFOA/PFOS in the United States and other countries.

State/country	PFOA (ngL^−1^)	PFOS (ngL^−1^)	Other PFAS	Source	Year

USEPA	70	70		USEPA	2016
Alabama	400	200		USEPA	2009
Alaska	70	70		AK DEC	2018
California	10	40	PFBS	CA SWRCB	2021
Connecticut	70	70	PFNA, PFHxS	CT DPH	2016
Delaware	70	70		DDREC	2017
Georgia	400	200		EPD	2009
IIllinois	2	14	PFBS, PFHxS, PFHxA	EPA	2021
Maine	70	70		ME DEP	2020
Massachusetts	20	20	PFNA, PFHxS, PFHpA, PFDA	MA DEP	2018/2019
Michigan	8	16	PFNA, PFBS, PFHxS, PFHxA, Gen-X	MI EGLE	2021
Minnesota	35	300	700 PFBA	MDH	2009
Nevada	667	667	PFBS	NV DEP	2015
New Hampshire	12	15	PFNA, PFHxS	NH DES	2020
New Jersey	14	13	PFNA	NJDEP	2020
New Mexico	70	70	PFHxS	NMED	2019
New York	10	10		NY DOH	2020
North Carolina	-	-	Gen-X	NC DHHS	2017
Ohio	70	70	PFNA, PFBS, PFHxS, Gen-X	ODH	2019
Pennsylvania	TBD	TBD		PA DEP	2015
Texas	100	100	PCLs for 16 PFAS	TCEQ	2013
Rhode Island	70	70		RI DEM	2017
Vermont	20	20	PFNA, PFHxS, PFHpA	VT DEC	2020
West Virginia	400	200		USEPA	2009
Australia	560	70		ADEH	2017
Canada	200	600		CAN HC	2018
Denmark	100	100	PFNA, PFBA/S, PFHxS/A, PFPeA, PFHpA, PFOSA, PFDA, 6:2 FTS	EPA	2015
Germany	300	300		GMOH	2006
Italy	500	30	PFBA, PFBS, PFHxA, PFPeA		2017
Netherlands	390	200	Gen-X	EPA	2020
Sweden	90	90	PFBS, PFHxS, PFHxA, PFPeA, PFHpA	NFA	2014
United Kingdom	10	10		UKDWI	2021

## HIGH LIGHTS

- In-depth review of worldwide PFAS pollution in water resources
- Description of novel and legacy PFAS; their sources, occurrence, and distribution
- Discussion of regulatory frameworks for PFAS concentrations in environmental media

## Abbreviations:

AFB: Air Force Base
AFFFs: Aqueous Film-Forming Foams
AGQS: Ambient Groundwater Quality Standard
ASTSWMO: Association of State And Territorial Solid Waste Management Officials
CCL: Contaminant Candidate List
C-F bond: Carbon-Fluorine bond
diPAPs: Polyfluoroalkyl Phosphate Diesters
EC: European Commission
ECF: Electrochemical Fluorination
EQS: Environmental Quality Standards
EtFOSA: Ethyl Perfluorooctane Sulfonamide
F53B: Chlorinated Polyfluorinated Ether Sulfonate
FPP: Fluorochemical Processing Plant
FTSA: Fluorotelomer Sulfonate
FOSA: Perfluorooctane Sulfonamide
FOSAA: Perfluorinated Sulfonamidoacetic Acids
FHxSA: Perfluorohexane Sulfonamide
HFPO: Hexafluoropropylene Oxide
ITRC: Interstate Technology and Regulatory Council
LC-MS/MS: Liquid Chromatography-tandem Mass Spectrometry
LHA: Lifetime Health Advisory
LOD: Limit of Detection
LOQ: Limit of Quantification
MDL: Method Detection Limit
MONET: MONitoring NETwork
MQL: Method Quantitation Limit
MS/MS: Tandem Mass Spectrometry (MS/MS)
ND: Non-detect
MCL: Maximum Contaminant Levels
NHDES: New Hampshire Department of Environmental Services
NJDEP: New Jersey Department of Environmental Protection
NLs: Notification Levels
OCWD: Orange County Water District
PFA: Perfluorinated Acid
PFAS: Per- and polyfluoroalkyl Substances
PFBA: Perfluorobutanoic Acid
PFBS: Perfluorobutanoic Sulfonate
PFCA: Perfluoroalkyl Carboxylic Acid
PFCs: Perfluorochemicals
PFECAs: Perfluoroalkyl Ether Carboxylic Acids
PFECHS: Perfluoroethylcyclohexanesulfonate
PFESA: Perfluoroalkyl Ether Sulfonic Acid
PFHxA: Perfluorohexanoic Acid
PFHxS: Perfluorohexane Sulfonic acid
PFOA: Perfluorooctanoic Acid
PFOS: Perfluorooctane Sulfonic Acid
PFOSAs: Perfluorooctane Sulfonamides
PFPEs: Perfluoropolyether
PFPeA: Perfluoropentanoic Acid
PFSA: Perfluoroalkyl Sulfonic Acids
POPs: Persistent Organic Pollutants
PFNA: Perfluorononanoic Acid
Q-TOF-MS: Quadrupole Time-of-Flight Mass Spectrometry
R: Retardation Factor
RLs: Response Levels
UCMR: Unregulated Contaminant Monitoring Rule
UNEP: United Nations Environment Programme
US EPA: United States Environmental Protection Agency
USGS: United States Geological Survey
WWTPs: Wastewater Treatment Plants
WDOE: Washington State Department of Ecology

## References

[R1] AFB, 2017. Preliminary Groundwater Sampling Results Indicate Contaminants in Airway Heights Water well. https://www.fairchild.af.mil/News/Article-Display/Article/1184640/preliminary-groundwater-sampling-results-indicate-contaminants-in-airway-height/. (Accessed 4 August 2021).

[R2] Ahrens L, 2011. Polyfluoroalkyl compounds in the aquatic environment: a review of their occurrence and fate. J. Environ. Monit 13, 20–31. 10.1039/C0EM00373E. 21031178

[R3] Ahrens L, Bundschuh M, 2014. Fate and effects of poly- and perfluoroalkyl substances in the aquatic environment: a review. Environ. Toxicol. Chem 33, 1921–1929. 10.1002/ETC.2663. 24924660

[R4] Aminot Y, Sayfritz SJ, Thomas KV, Godinho L, Botteon E, Ferrari F, Boti V, Albanis T, Köck-Schulmeyer M, Diaz-Cruz MS, Farré M, Barceló D, Marques A, Readman JW, 2019. Environmental risks associated with contaminants of legacy and emerging concern at european aquaculture areas. Environ. Pollut 252, 1301–1310. 10.1016/J.ENVPOL.2019.05.133. 31252127

[R5] Anderson JK, Luz AL, Goodrum P, Durda J, 2019. Perfluorohexanoic acid toxicity, part II: application of human health toxicity value for risk characterization. Regul. Toxicol. Pharmacol 103, 10–20. 10.1016/J.YRTPH.2019.01.020. 30634020

[R6] Astswmo, 2015. Perfluorinated Chemicals (PFCs): PFOA & PFOS Information Paper – ASTSWMO [WWW Document]. https://astswmo.org/perfluorinated-chemicals-pfcs-pfoa-pfos-information-paper/ accessed 7.30.20.

[R7] Baabish A, Sobhanei S, Fiedler H, 2021. Priority perfluoroalkyl substances in surface waters - a snapshot survey from 22 developing countries. Chemosphere 273, 129612. 10.1016/J.CHEMOSPHERE.2021.129612. 33493820

[R8] Bai X, Son Y, 2021. Perfluoroalkyl substances (PFAS) in surface water and sediments from two urban watersheds in Nevada, USA. Sci. Total Environ 751, 141622. 10.1016/J.SCITOTENV.2020.141622. 32871315

[R9] Banzhaf S, Filipovic M, Lewis J, Sparrenbom CJ, Barthel R, 2016. A review of contamination of surface-, ground-, and drinking water in Sweden by perfluoroalkyl and polyfluoroalkyl substances (PFASs). Ambio 463 (46), 335–346. 10.1007/S13280-016-0848-82016. PMC5347527 27844420

[R10] Bao J, Liu W, Liu L, Jin Y, Dai J, Ran X, Zhang Z, Tsuda S, 2011. Perfluorinated Compounds in the Environment and the Blood of Residents Living near Fluorochemical Plants in FuxinChina. 45, 8075–8080. 10.1021/ES102610X. 20964291

[R11] Barceló D, Ruan T, 2019. Challenges and perspectives on the analysis of traditional perfluoroalkyl substances and emerging alternatives. TrAC Trends Anal. Chem 121, 115605. 10.1016/J.TRAC.2019.07.016.

[R12] Benskin JP, Muir DCG, Scott BF, Spencer C, Silva AO De, Kylin H, Martin JW, Morris A, Lohmann R, Tomy G, Rosenberg B, Taniyasu S, Yamashita N, 2012. Perfluoroalkyl acids in the Atlantic and Canadian arctic oceans. Environ. Sci. Technol 46, 5815–5823. 10.1021/ES300578X. 22548373

[R13] Boone JS, Vigo C, Boone T, Byrne C, Ferrario J, Benson R, Donohue J, Simmons JE, Kolpin DW, Furlong ET, Glassmeyer ST, 2019. Per- and polyfluoroalkyl substances in source and treated drinking waters of the United States. Sci. Total Environ 653, 359–369. 10.1016/J.SCITOTENV.2018.10.245. 30412881 PMC6996027

[R14] Boulanger B, Vargo J, Schnoor JL, Hornbuckle KC, 2004. Detection of perfluorooctane surfactants in Great Lakes water. Environ. Sci. Technol 38, 4064–4070. 10.1021/ES0496975. 15352442

[R15] Bräunig J, Baduel C, Heffernan A, Rotander A, Donaldson E, Mueller JF, 2017. Fate and redistribution of perfluoroalkyl acids through AFFF-impacted groundwater. Sci. Total Environ 596–597, 360–368. 10.1016/J.SCITOTENV.2017.04.095. 28441576

[R16] Buck RC, Franklin J, Berger U, Conder JM, Cousins IT, Voogt P De, Jensen AA, Kannan K, Mabury SA, van Leeuwen SPJ, 2011. Perfluoroalkyl and polyfluoroalkyl substances in the environment: Terminology, classification, and origins. Integr. Environ. Assess. Manag 7, 513–541. 10.1002/IEAM.258. 21793199 PMC3214619

[R17] Buck RC, Murphy PM, Pabon M, 2012. Chemistry, properties, and uses of commercial fluorinated surfactants. Handb. Environ. Chem 17, 1–24. 10.1007/978-3-642-21872-9_1.

[R18] Calafat AM, Wong LY, Kuklenyik Z, Reidy JA, Needham LL, 2007. Polyfluoroalkyl chemicals in the U.S. population: data from the national health and nutrition examination survey (NHANES) 2003–2004 and comparisons with NHANES 1999–2000. Environ. Health Perspect 115, 1596–1602. 10.1289/EHP.10598. 18007991 PMC2072821

[R19] Campo J, Pérez F, Masiá A, Picó Y, la Farré M, Barceló D, 2015. Perfluoroalkyl substance contamination of the Llobregat River ecosystem (Mediterranean area, NE Spain). Sci. Total Environ 503–504, 48–57. 10.1016/J.SCITOTENV.2014.05.094. 24935262

[R20] Campo J, Lorenzo M, Pérez F, Picó Y, la Farré M, Barceló D, 2016. Analysis of the presence of perfluoroalkyl substances in water, sediment and biota of the Jucar River (E Spain). Sources, partitioning and relationships with water physical characteristics. Environ. Res 147, 503–512. 10.1016/J.ENVRES.2016.03.010. 26974364

[R21] Chen C, Lu Y, Zhang X, Geng J, Wang T, Shi Y, Hu W, Li J, 2009. A review of spatial and temporal assessment of PFOS and PFOA contamination in China. 25, pp. 163–177. 10.1080/02757540902918321 10.1080/02757540902918321.

[R22] Chen H, Zhang L, Li M, Yao Y, Zhao Z, Munoz G, Sun H, 2019. Per- and polyfluoroalkyl substances (PFASs) in precipitation from mainland China: contributions of unknown precursors and short-chain (C2C3) perfluoroalkyl carboxylic acids. Water Res. 153, 169–177. 10.1016/J.WATRES.2019.01.019. 30711792

[R23] Cheremisinoff NP, 2017. Perfluorinated Chemicals (PFCs) - Contaminants of Concern. December 2016. John Wiley & Sons 978–1-119–36353-8.

[R24] Choi GH, Lee DY, Jeong DK, Kuppusamy S, Lee YB, Park BJ, Kim JH, 2017. Perfluorooctanoic acid (PFOA) and perfluorooctanesulfonic acid (PFOS) concentrations in the South Korean agricultural environment: a national survey. J. Integr. Agric 16, 1841–1851. 10.1016/S2095-3119(16)61585-X.

[R25] Chu S, Letcher RJ, 2017. Side-chain fluorinated polymer surfactants in aquatic sediment and biosolid-augmented agricultural soil from the Great Lakes basin of North America. Sci. Total Environ 607–608, 262–270. 10.1016/J.SCITOTENV.2017.06.252. 28692896

[R26] Corsini E, Luebke RW, Germolec DR, DeWitt JC, 2014. Perfluorinated compounds: emerging POPs with potential immunotoxicity. Toxicol. Lett 230, 263–270. 10.1016/J.TOXLET.2014.01.038. 24503008 PMC4439925

[R27] Crone BC, Speth TF, Wahman DG, Smith SJ, Abulikumu G, Kleiner EJ, Pressman JG, 2019. Occurrence of per- and polyfluoroalkyl substances (PFAS) in source water and their treatment in drinking water. Crit. Rev. Environ. Sci. Technol 49 (24), 2359–2396. 10.1080/10643389.2019.1614848. PMC7433796 32831535

[R28] Cui D, Li X, Quinete N, 2020. Occurrence, fate, sources and toxicity of PFAS: what we know so far in Florida and major gaps. TrAC Trends Anal. Chem 130, 115976. 10.1016/J.TRAC.2020.115976.

[R29] D’Agostino LA, Mabury SA, 2017. Certain perfluoroalkyl and polyfluoroalkyl substances associated with aqueous film forming foam are widespread in Canadian surface waters. Environ. Sci. Technol 51, 13603–13613. 10.1021/ACS.EST.7B03994. 29110476

[R30] Dean WS, Adejumo HA, Caiati A, Garay PM, Harmata AS, Li L, Rodriguez EE, Sundar S, 2020. A Framework for Regulation of New and Existing PFAS by EPA. J. Sci. Policy Gov. POLICY Anal. Regul. PFAS BY EPA www.sciencepolicyjournal.org JSPG 16.

[R31] Díaz-Cruz MS, García-Galán MJ, Guerra P, Jelic A, Postigo C, Eljarrat E, Farré M, López de Alda MJ, Petrovic M, Barceló D, Petrovic M, 2009. Analysis of selected emerging contaminants in sewage sludge. TrAC Trends Anal. Chem 28, 1263–1275. 10.1016/J.TRAC.2009.09.003.

[R32] Du Z, Deng S, Bei Y, Huang Q, Wang B, Huang J, Yu G, 2014. Adsorption behavior and mechanism of perfluorinated compounds on various adsorbents—a review. J. Hazard. Mater 274, 443–454. 10.1016/J.JHAZMAT.2014.04.038. 24813664

[R33] Duong HT, Kadokami K, Shirasaka H, Hidaka R, Chau HTC, Kong L, Nguyen TQ, Nguyen TT, 2015. Occurrence of perfluoroalkyl acids in environmental waters in Vietnam. Chemosphere 122, 115–124. 10.1016/J.CHEMOSPHERE.2014.11.023. 25496738

[R34] DuPont TM, 2010. DuPontTM GenX processing aid for making fluoropolymer resins; setting a new industry standard for sustainable replacement technology.

[R35] EPA, 2020. PFAS Action Plan. https://www.epa.gov/sites/default/files/2020-01/documents/pfas_action_plan_feb2020.pdf. (Accessed 5 August 2021).

[R36] Espana VA, Mallavarapu M, Naidu R, 2015. Treatment technologies for aqueous perfluorooctanesulfonate (PFOS) and perfluorooctanoate (PFOA): A critical review with an emphasis on field testing. Environ. Technol. Innov 4, 168–181. 10.1016/J.ETI.2015.06.001.

[R37] Exner M, Färber H, 2006. Perfluorinated surfactants in surface and drinking waters (9 pp). Environ. Sci. Pollut. Res 135 (13), 299–307. 10.1065/ESPR2006.07.326. 17067024

[R38] Fernandez NA, Rodriguez-Freire L, Keswani M, Sierra-Alvarez R, 2016. Effect of chemical structure on the sonochemical degradation of perfluoroalkyl and polyfluoroalkyl substances (PFASs). Environ. Sci. Water Res. Technol 2, 975–983. 10.1039/C6EW00150E.

[R39] Furdui VI, Crozier PW, Reiner EJ, Mabury SA, 2008. Trace level determination of perfluorinated compounds in water by direct injection. Chemosphere 73, S24–S30. 10.1016/J.CHEMOSPHERE.2007.07.085. 18457864

[R40] Furl C, Meredith C, 2010. Perfluorinated Compounds in Washington Rivers and Lakes. Toxics Studies Unit Environmental Assessment Program, Washington State Department of Ecology, Olympia, Washington 98504–7710. https://apps.ecology.wa.gov/publications/documents/1003034.pdf.

[R41] Gallen C, Drage D, Eaglesham G, Grant S, Bowman M, Mueller JF, 2017. Australia-wide assessment of perfluoroalkyl substances (PFASs) in landfill leachates. J. Hazard. Mater 331, 132–141. 28254660 10.1016/j.jhazmat.2017.02.006

[R42] Gebbink WA, van Leeuwen SPJ, 2020. Environmental contamination and human exposure to PFASs near a fluorochemical production plant: Review of historic and current PFOA and GenX contamination in the Netherlands. Environ. Int 137, 105583. 10.1016/J.ENVINT.2020.105583. 32106048

[R43] Gebbink WA, van Asseldonk L, van Leeuwen SPJ, 2017. Presence of emerging per- and polyfluoroalkyl substances (PFASs) in river and drinking water near a fluorochemical production plant in the Netherlands. Environ. Sci. Technol 51, 11057–11065. 10.1021/ACS.EST.7B02488. PMC5677760 28853567

[R44] Ghisi R, Vamerali T, Manzetti S, 2019. Accumulation of perfluorinated alkyl substances (PFAS) in agricultural plants: a review. Environ. Res 169, 326–341. 10.1016/J.ENVRES.2018.10.023. 30502744

[R45] Gilljam JL, Leonel J, Cousins IT, Benskin JP, 2015. Is ongoing sulfluramid usein South America a significant source of perfluorooctanesulfonate (PFOS)? Production inventories, environmental fate, and local occurrence. Environ. Sci. Technol 50, 653–659. 10.1021/ACS.EST.5B04544. 26653085

[R46] Glüge J, Scheringer M, Cousins IT, DeWitt JC, Goldenman G, Herzke D, Lohmann R, Ng CA, Trier X, Wang Z, 2020. An overview of the uses of per- and polyfluoroalkyl substances (PFAS). Environ. Sci. Process. Impacts 22, 2345–2373. 10.1039/D0EM00291G. 33125022 PMC7784712

[R47] Göckener B, Fliednerb A, Rüdel H, Fettig I, Koschorreck J, 2021. Exploring unknown per- and polyfluoroalkyl substances in the German environment – the total oxidizable precursor assay as helpful tool in research and regulation. Sci. Total Environ 782, 146825. 10.1016/j.scitotenv.2021.146825. 33838381

[R48] Goodrow SM, Ruppel B, Lippincott RL, Post GB, Procopio NA, 2020. Investigation of levels of perfluoroalkyl substances in surface water, sediment and fish tissue in New Jersey, USA. Sci. Total Environ 729, 138839. 10.1016/J.SCITOTENV.2020.138839. 32387771

[R49] Goverment NSW, 2015. Preliminary Surface Water Investigation Results – Williamtown NSW [WWW Document]. https://www.epa.nsw.gov.au/-/media/epa/corporate-site/resources/epa/williamtown-preliminary-surface-water-results.pdf?la=en&hash=9C01B53598330584777940884A6C1AC39A17D12C. (Accessed 30 July 2021).

[R50] Grandjean P, Andersen EW, Budtz-Jørgensen E, Nielsen F, Mølbak K, Weihe P, Heilmann C, 2012. Serum vaccine antibody concentrations in children exposed to perfluorinated compounds. JAMA 307, 391–397. 10.1001/JAMA.2011.2034. 22274686 PMC4402650

[R51] Groffen T, Wepener V, Malherbe W, Bervoets L, 2018. Distribution of perfluorinated compounds (PFASs) in the aquatic environment of the industrially polluted Vaal RiverSouth Africa. 627, 1334–1344. 10.1016/j.scitotenv.2018.02.023 30857097

[R52] Houde M, Silva AO De, Muir DCG, Letcher RJ, 2011. Monitoring of. Environ. Sci. Technol 45, 7962–7973. 10.1021/ES104326W. 21542574

[R53] Hu XC, Andrews DQ, Lindstrom AB, Bruton TA, Schaider LA, Grandjean P, Lohmann R, Carignan CC, Blum A, Balan SA, Higgins CP, Sunderland EM, 2016. Detection of poly- and perfluoroalkyl substances (PFASs) in U.S. drinking water linked to industrial sites, military fire training areas, and wastewater treatment plants. Environ. Sci. Technol. Lett 3, 344–350. 10.1021/ACS.ESTLETT.6B00260. 27752509 PMC5062567

[R54] ITRC, 2020. Naming Conventions for Per- and Polyfluoroalkyl Substances (PFAS) [WWW Document]. https://pfas-dev.itrcweb.org/wp-content/uploads/2020/10/naming_conventions_508_2020Aug_Final.pdf. (Accessed 30 July 2021).

[R55] Janousek RM, Mayer J, Knepper TP, 2019. Is the phase-out of long-chain PFASs measurable as fingerprint in a defined area? Comparison of global PFAS concentrations and a monitoring study performed in Hesse, Germany from 2014 to 2018. TrAC Trends Anal. Chem 120, 115393.

[R56] Jian JM, Guo Y, Zeng L, Liang-Ying L, Lu X, Wang F, Zeng EY, 2017. Global distribution of perfluorochemicals (PFCs) in potential human exposure source–a review. Environ. Int 108, 51–62. 10.1016/J.ENVINT.2017.07.024. 28800414

[R57] Jin YH, Liu W, Sato I, Nakayama SF, Sasaki K, Saito N, Tsuda S, 2009. PFOS and PFOA in environmental and tap water in China. Chemosphere 77, 605–611. 10.1016/J.CHEMOSPHERE.2009.08.058. 19775722

[R58] Kaboré HA, Vo Duy S, Munoz G, Méité L, Desrosiers M, Liu J, Sory TK, Sauvé S, 2018. Worldwide drinking water occurrence and levels of newly-identified perfluoroalkyl and polyfluoroalkyl substances. Sci. Total Environ 616–617, 1089–1100. 10.1016/J.SCITOTENV.2017.10.210. 29100694

[R59] KEMI, 2015. Occurrence and use of highly fluorinated substances and alternatives Report from a government assignment.

[R60] Kucharzyk KH, Darlington R, Benotti M, Deeb R, Hawley E, 2017. Novel treatment technologies for PFAS compounds: a critical review. J. Environ. Manag 204, 757–764. 10.1016/J.JENVMAN.2017.08.016. 28818342

[R61] Kunacheva C, Fujii S, Tanaka S, Seneviratne STMLD, Lien NPH, Nozoe M, Kimura K, Shivakoti BR, Harada H, 2012. Worldwide surveys of perfluorooctane sulfonate (PFOS) and perfluorooctanoic acid (PFOA) in water environment in recent years. Water Sci. Technol 66, 2764–2771. 10.2166/WST.2012.518. 23109596

[R62] Lam NH, Cho C, Kannan K, Cho H, 2017. A nationwide survey of perfluorinated alkyl substances in waters, sediment and biota collected from aquatic environment in Vietnam: distributions and bioconcentration profiles. J. Hazard. Mater 323, 116–127. 10.1016/j.jhazmat.2016.04.010. 27106518

[R63] Li F, Zhang C, Qu Y, Chen J, Chen L, Liu Y, Zhou Q, 2010. Quantitative characterization of short- and long-chain perfluorinated acids in solid matrices in ShanghaiChina. 408, 617–623. 10.1016/J.SCITOTENV.2009.10.032. 19896166

[R64] Li Q, Wang T, Zhu Z, Meng J, Wang P, Suriyanarayanan S, Zhang Y, Zhou Y, Song S, Lu Y, Yvette B, 2017. Using hydrodynamic model to predict PFOS and PFOA transport in the Daling River and its tributary, a heavily polluted river into the Bohai Sea, China. Chemosphere 167, 344–352. 10.1016/J.CHEMOSPHERE.2016.09.119. 27741427

[R65] Lindstrom AB, Strynar MJ, Delinsky AD, Nakayama SF, McMillan L, Libelo EL, Neill M, Thomas L, 2011. Application of WWTP Biosolids and Resulting Perfluorinated Compound Contamination of Surface and Well Water in Decatur, AlabamaUSA. 45, 8015–8021. 10.1021/ES1039425. 21513287

[R66] Liu Y, Qian M, Ma X, Zhu L, Martin JW, 2018. Nontarget Mass Spectrometry Reveals New Perfluoroalkyl Substances in Fish from the Yangtze River and Tangxun LakeChina. 52, 5830–5840. 10.1021/ACS.EST.8B00779. 29659273

[R67] Llorca M, Farré M, Picó Y, Müller J, Knepper TP, Barceló D, 2012. Analysis of perfluoroalkyl substances in waters from Germany and Spain. Sci. Total Environ 431, 139–150. 10.1016/J.SCITOTENV.2012.05.011. 22683491

[R68] Lorenzo M, Campo J, Farré M, Pérez F, Picó Y, Barceló D, 2016. Perfluoroalkyl substances in the Ebro and Guadalquivir river basins (Spain). Sci. Total Environ 540, 191–199. 10.1016/j.scitotenv.2015.07.045. 26250865

[R69] Lu Z, Song L, Zhao Z, Ma Y, Wang J, Yang H, Ma H, Cai M, Codling G, Ebinghaus R, Xie Z, Giesy JP, 2015. Occurrence and trends in concentrations of perfluoroalkyl substances (PFASs) in surface waters of eastern China. Chemosphere 119, 820–827. 10.1016/J.CHEMOSPHERE.2014.08.045. 25218980

[R70] Mahinroosta R, Senevirathna L, 2020. A review of the emerging treatment technologies for PFAS contaminated soils. J. Environ. Manag 255, 109896. 10.1016/J.JENVMAN.2019.109896. 32063301

[R71] Marchiandi J, Szabo D, Dagnino S, Green MP, Clarke BO, 2021. Occurrence and fate of legacy and novel per- and polyfluoroalkyl substances (PFASs) in freshwater after an industrial fire of unknown chemical stockpiles. Environ. Pollut 278, 116839. 10.1016/J.ENVPOL.2021.116839. 33740602

[R72] McLachlan MS, Holmström KE, Reth M, Berger U, 2007. Riverine discharge of perfluorinated carboxylates from the European continent. Environ. Sci. Technol 41, 7260–7265. 10.1021/ES071471P. 18044497

[R73] Moody CA, Field JA, 2020. Perfluorinated surfactants and the environmental implications of their use in fire-fighting foams. Environ. Sci. Technol 34 (18), 1544–1552. 10.1021/es991359u.

[R74] Munoz G, Labadie P, Botta F, Lestremau F, Lopez B, Geneste E, Pardon P, Dévier MH, Budzinski H, 2017. Occurrence survey and spatial distribution of perfluoroalkyl and polyfluoroalkyl surfactants in groundwater, surface water, and sediments from tropical environments. Sci. Total Environ 607–608, 243–252. 10.1016/J.SCITOTENV.2017.06.146. 28692894

[R75] Nakayama SF, Strynar MJ, Reiner JL, Delinsky AD, Lindstrom AB, 2010. Determination of perfluorinated compounds in the Upper Mississippi River basin. Environ. Sci. Technol 44, 4103–4109. 10.1021/ES100382Z. 20441143

[R76] Newell CJ, Adamson DT, Kulkarni PR, Nzeribe BN, Stroo H, 2020. Comparing PFAS to other groundwater contaminants: implications for remediation. Remediat. J 30, 7–26. 10.1002/REM.21645.

[R77] Newton S, McMahen R, Stoeckel JA, Chislock M, Lindstrom A, Strynar M, 2017. Novel Polyfluorinated Compounds Identified Using High Resolution Mass Spectrometry Downstream of Manufacturing Facilities near DecaturAlabama. 51, 1544–1552. 10.1021/ACS.EST.6B05330. PMC6620606 28084732

[R78] Nguyen TV, Reinhard M, Chen H, Gin KY-H, 2016. Fate and transport of perfluoro- and polyfluoroalkyl substances including perfluorooctane sulfonamides in a managed urban water body. Environ. Sci. Pollut. Res 2311 (23), 10382–10392. 10.1007/S11356-016-6788-9. 27146547

[R79] NHDES, 2021. Status Report on the Occurrence of Per- and Polyfluoroalkyl Substancecs (PFAS) Contamination in New Hampshire. https://www4.des.state.nh.us/nh-pfas-investigation/wp-content/uploads/Statewide-PFAS-Occurrence-Status-Report.June_.2021.pdf.

[R80] Nicole W, 2013. PFOA and cancer in a highly exposed community: new findings from the C8 science panel. Environ. Health Perspect 121, A340. 10.1289/EHP.121-A340. 24284021 PMC3855507

[R81] NJDEP, 2014. Occurrence of Perfluorinated Chemicals in Untreated New Jersey Drinking Water Sources Final Report. https://www.nj.gov/dep/watersupply/pdf/pfc-study.pdf.

[R82] North Carolina Department of Health and Human Services, 2018. Biological sampling for GenX and other Per-and Polyfluoroalkyl Substances (PFAS)-North Carolina, 2018 Summary. https://epi.dph.ncdhhs.gov/oee/pfas/NCDHHS_PFAS%20Biomonitoring%20Report_8Nov2018.pdf.

[R83] OCWD, 2016. Ensuring Groundwater and Drinking Water Supplies Meet EPA’s Health Advisory for PFOA and PFOS, Background on Unregulated Chemicals:PFOA and PFOS, Factsheet. on 10/21/2017 https://www.ocwd.com/media/4376/pfoa-pfos-fact-sheet_v41-4pg.pdf.

[R84] OCWD (Orange County Water District), 2021. PFAS in Orange Count. What Are They, how do they impact us and what’s being done, pp. 1–2.

[R85] Oliaei F, Kriens D, Weber R, Watson A, 2013. PFOS and PFC releases and associated pollution from a PFC production plant in Minnesota (USA). Environ. Sci. Pollut. Res 204 (20), 1977–1992. 10.1007/S11356-012-1275-42012. 23128989

[R86] Pan Y, Zhang H, Cui Q, Sheng N, Yeung LWY, Sun Y, Guo Y, Dai J, 2018. Worldwide distribution of novel perfluoroether carboxylic and sulfonic acids in surface water. Environ. Sci. Technol 52, 7621–7629. 10.1021/ACS.EST.8B00829. 29749740

[R87] Parsons JR, Sáez M, Dolfing J, de Voogt P, 2008. Biodegradation of perfluorinated compounds. Rev. Environ. Contam. Toxicol 196, 53–71. 10.1007/978-0-387-78444-1_2. 19025092

[R88] Pignotti E, Casas G, Llorca M, Tellbüscher A, Almeida D, Dinelli E, Farré M, Barceló D, 2017. Seasonal variations in the occurrence of perfluoroalkyl substances in water, sediment and fish samples from Ebro Delta (Catalonia, Spain). Sci. Total Environ 607–608, 933–943. 10.1016/J.SCITOTENV.2017.07.025. 28724225

[R89] Plumlee MH, Larabee J, Reinhard M, 2008. Perfluorochemicals in water reuse. Chemosphere 72, 1541–1547. 10.1016/J.CHEMOSPHERE.2008.04.057. 18547612

[R90] Quinete N, Wu Q, Zhang T, Yun SH, Moreira I, Kannan K, 2009. Specific profiles of perfluorinated compounds in surface and drinking waters and accumulation in mussels, fish, and dolphins from southeastern Brazil. Chemosphere 77, 863–869. 10.1016/J.CHEMOSPHERE.2009.07.079. 19744696

[R91] Rahman MF, Peldszus S, Anderson WB, 2014. Behaviour and fate of perfluoroalkyl and polyfluoroalkyl substances (PFASs) in drinking water treatment: a review. Water Res. 50, 318–340. 10.1016/J.WATRES.2013.10.045. 24216232

[R92] Rauert C, Harner T, Schuster JK, Eng A, Fillmann G, Castillo LE, Fentanes O, Ibarra MV, Miglioranza KSB, Rivadeneira IM, Pozo K, Zuluaga BHA, 2018. Atmospheric concentrations of new persistent organic pollutants and emerging chemicals of concern in the group of Latin America and Caribbean (GRULAC) region. Environ. Sci. Technol 52, 7240–7249. 10.1021/ACS.EST.8B00995. 29846065

[R93] Rayne S, Forest K, 2009. Perfluoroalkyl sulfonic and carboxylic acids: A critical review of physicochemical properties, levels and patterns in waters and wastewaters, and treatment methods. 44, 1145–1199. 10.1080/10934520903139811 doi:10.1080/10934520903139811. 19847705

[R94] Ruyle BJ, Pickard HM, LeBlanc DR, Tokranov AK, Thackray CP, Hu XC, Vecitis CD, Sunderland EM, 2021. Isolating the AFFF signature in coastal watersheds using oxidizable PFAS precursors and unexplained organofluorine. Environ. Sci. Technol 55, 3686–3695. 10.1021/ACS.EST.0C07296. 33667081 PMC11082878

[R95] Sammut G, Sinagra E, Helmus R, de Voogt P, 2017. Perfluoroalkyl substances in the Maltese environment – (I) surface water and rain water. Sci. Total Environ 589, 182–190. 10.1016/J.SCITOTENV.2017.02.128. 28259432

[R96] Scher DP, Kelly JE, Huset CA, Barry KM, Hoffbeck RW, Yingling VL, Messing RB, 2018. Occurrence of perfluoroalkyl substances (PFAS) in garden produce at homes with a history of PFAS-contaminated drinking water. Chemosphere 196, 548–555. 10.1016/J.CHEMOSPHERE.2017.12.179. 29329087

[R97] Schulz K, Silva MR, Klaper R, 2020. Distribution and effects of branched versus linear isomers of PFOA, PFOS, and PFHxS: a review of recent literature. Sci. Total Environ 733, 139186. 10.1016/J.SCITOTENV.2020.139186. 32474294

[R98] Schwanz TG, Llorca M, Farré M, Barceló D, 2016. Perfluoroalkyl substances assessment in drinking waters from BrazilFrance and Spain. 539, 143–152. 10.1016/j.scitotenv.2015.08.034 26360456

[R99] Sharifan H, Bagheri M, Wang D, Burken JG, Higgins CP, Liang Y, Liu J, Schaefer CE, Blotevogel J, 2021. Fate and transport of per- and polyfluoroalkyl substances (PFASs) in the vadose zone. Sci. Total Environ 771, 145427. 10.1016/J.SCITOTENV.2021.145427. 33736164

[R100] Sharma BM, Bharat GK, Tayal S, Larssen T, Bečanová J, Karásková P, Whitehead PG, Futter MN, Butterfield D, Nizzetto L, 2016. Perfluoroalkyl substances (PFAS) in river and ground/drinking water of the Ganges River basin: emissions and implications for human exposure. Environ. Pollut 208, 704–713. 10.1016/J.ENVPOL.2015.10.050. 26561452

[R101] Silva AO De, Spencer, Scott BF, Backus S, Muir DCG, 2011. Detection of a cyclic perfluorinated acid, perfluoroethylcyclohexane sulfonate, in the Great Lakes of North America. Environ. Sci. Technol 45, 8060–8066. 10.1021/ES200135C. 21528907

[R102] Sindiku O, Orata F, Weber R, Osibanjo O, 2013. Per- and polyfluoroalkyl substances in selected sewage sludge in Nigeria. Chemosphere 92, 329–335. 10.1016/J.CHEMOSPHERE.2013.04.010. 23648329

[R103] Skutlarek D, Exner M, Farber H, 2006. Perfluorinated surfactants in surface and drinking water. Environ. Sci. Pollut. Res 13, 299–307. 10.1065/espr2006.07.326 17067024

[R104] Sleep JA, Juhasz AL, 2020. Perfluoroalkyl, fluorotelomer sulfonate, and perfluorooctane sulfonamide contamination in biosolids: Composition, co-contamination and re-use implications. Environ. Pollut 266. 10.1016/j.envpol.2020.115120 32682161

[R105] So MK, Miyake Y, Yeung WY, Ho YM, Taniyasu S, Rostkowski P, Yamashita N, Zhou BS, Shi XJ, Wang JX, Giesy JP, Yu H, Lam PKS, 2007. Perfluorinated compounds in the Pearl River and Yangtze River of China. 68, pp. 2085–2095. 10.1016/j.chemosphere.2007.02.008. 17368725

[R106] Stefanac T, McCrindle R, McAlees AJ, Riddell N, Brazeau AL, Chittim BC, 2018. Characterization of nine isomers in commercial samples of perfluoroethylcyclohexanesulfonate and of some minor components including PFOS isomers. Environ. Sci. Technol 52, 9937–9945. 10.1021/ACS.EST.8B02369. 30063347

[R107] Stock NL, Furdui VI, Muir DCG, Mabury SA, Stock Naomi L., Furdui Vasile I., Muir Derek C.G., Mabury Scott A., 2007. Perfluoroalkyl contaminants in the Canadian Arctic: evidence of atmospheric transport and local contamination. Environ. Sci. Technol 41 (10), 3529–3536. 10.1021/ES062709X. 17547174

[R108] Stoiber T, Evans S, Naidenko OV, 2020. Disposal of products and materials containing per- and polyfluoroalkyl substances (PFAS): a cyclical problem. Chemosphere 260, 127659. 10.1016/J.CHEMOSPHERE.2020.127659. 32698118

[R109] Sunderland EM, Hu XC, Dassuncao C, Tokranov AK, Wagner CC, Allen JG, 2019. A review of the pathways of human exposure to poly- and perfluoroalkyl substances (PFASs) and present understanding of health effects. J. Expo. Sci. Environ. Epidemiol 29, 131–147. 10.1038/s41370-018-0094-1. 30470793 PMC6380916

[R110] Szabo D, Coggan TL, Robson TC, Currell M, Clarke BO, 2018. Investigating recycled water use as a diffuse source of per- and polyfluoroalkyl substances (PFASs) to groundwater in MelbourneAustralia. 644, 1409–1417. 10.1016/J.SCITOTENV.2018.07.048. 30743853

[R111] Tarapore P, Ouyang B, 2021. Perfluoroalkyl chemicals and male reproductive health: do PFOA and PFOS increase risk for male infertility? Int. J. Environ. Res. Public Health 18, 3794. 10.3390/ijerph18073794. 33916482 PMC8038605

[R112] UNEP, 2015. Global Monitoring Plan for Persistent Organic Pollutants Under the Stockholm Convenion Article 16 on Effectiveness Evaluation, Second Regional Monitoring Report, Afric Region. http://chm.pops.int/portals/0/download.aspx?d=UNEP-POPS-GMP-RMR-AFRICA-2015.English.pdf. (Accessed 14 July 2021).

[R113] UNEP, 2017. Global Monitoring Plan for Persistent Organic Pollutants Under the Stockholm Convention Article 16 on Effectiveness Evaluation 1–129. http://chm.pops.int/Portals/0/download.aspx?d=UNEP-POPS-COP.8-INF-38.English.pdf.

[R114] UNEP, 2019. Draft risk management evaluation: perfluorohexane sulfonic acid (PFHxS), its salts and PFHxS-related compounds. Persistent Organic Pollutants Review Committee, Fifteenth meeting Rome, 1–4 October 2019, Stockholm Convention on Persistent Organic Pollutants http://chm.pops.int/TheConvention/POPsReviewCommittee/Meetings/POPRC15/MeetingDocuments/tabid/8059/ctl/Download/mid/22056/Default.aspx?id=19&ObjID=27068.

[R115] UNEP, 2020. Acceptable Purposes:PFOS, Its Salts and PFOSF. http://chm.pops.int/Implementation/Exemptionsandacceptablepurposes/RegistersofAcceptablePurposes/AcceptablePurposesPFOSandPFOSF/tabid/794/Default.aspx. (Accessed 26 August 2021).

[R116] USEPA, 2016. Fact sheet PFOA & PFOS Drinking Water Health Advisories.

[R117] USEPA, 2017. Technical Fact Sheet – Perfluorooctane Sulfonate (PFOS) and Perfluorooctanoic Acid (PFOA).

[R118] USEPA, 2018. Fact Sheet: Draft Toxicity Assessments for GenX Chemicals and PFBS. https://www.epa.gov/sites/default/files/2018-11/documents/factsheet_pfbs-genxtoxicity_values_11.14.2018.pdf. (Accessed 2 August 2021).

[R119] Vo HNP, Ngo HN, Guo W, Nguyen TMH, Li J, Liang H, Deng L, Chen Z, Nguyen TAH, 2020. Poly-and perfluoroalkyl substances in water and wastewater: A comprehensive review from sources to remediation. J. Water Process Eng 36, 101393. 10.1016/J.JWPE.2020.101393.

[R120] Wang B, Cao M, Zhu H, Chen J, Wang L, Liu G, Gu X, Lu X, 2013. Distribution of perfluorinated compounds in surface water from Hanjiang River in Wuhan, China. Chemosphere 93, 468–473. 10.1016/J.CHEMOSPHERE.2013.06.014. 23830115

[R121] Wang Q, Ruan Y, Lin H, Lam PKS, 2020. Review on perfluoroalkyl and polyfluoroalkyl substances (PFASs) in the Chinese atmospheric environment. Sci. Total Environ 737, 139804. 10.1016/J.SCITOTENV.2020.139804. 32526580

[R122] Wang S, Ma L, Chen C, Li Y, Wu Y, Liu Y, Dou Z, Yamazaki E, Yamashita N, Lin B. Le, Wang X, 2020. Occurrence and partitioning behavior of per- and polyfluoroalkyl substances (PFASs) in water and sediment from the Jiulong Estuary-Xiamen Bay, China. Chemosphere 238. 10.1016/j.chemosphere.2019.124578 31524601

[R123] Wang T, Wang P, Meng J, Liu S, Lu Y, Khim JS, Giesy JP, 2015. A review of sources, multimedia distribution and health risks of perfluoroalkyl acids (PFAAs) in China. Chemosphere 129, 87–99. 10.1016/J.CHEMOSPHERE.2014.09.021. 25262946

[R124] Wang T, Vestergren R, Herzke D, Yu J, Cousins IT, 2016. Levels, isomer profiles, and estimated riverine mass discharges of perfluoroalkyl acids and fluorinated alternatives at the mouths of Chinese rivers. Environ. Sci. Technol 50, 11584–11592. 10.1021/ACS.EST.6B03752. 27689437

[R125] Wang X, Halsall C, Codling G, Xie Z, Xu B, Zhao Z, Xue Y, Ebinghaus R, Jones KC, 2013. Accumulation of perfluoroalkyl compounds in Tibetan Mountain snow: temporal patterns from 1980 to 2010. Environ. Sci. Technol 48, 173–181. 10.1021/ES4044775. 24320138

[R126] Wang Z, DeWitt JC, Higgins CP, Cousins IT, 2017. A never-ending story of per- and polyfluoroalkyl substances (PFASs)? Environ. Sci. Technol 51, 2508–2518. 10.1021/ACS.EST.6B04806. 28224793

[R127] Wanninayake DM, 2021. Comparison of currently available PFAS remediation technologies in water: a review. J. Environ. Manag 283, 111977. 10.1016/J.JENVMAN.2021.111977. 33517051

[R128] Wdoe, 2010. Perfluorinated Compounds in Washington Rivers and Lakes [WWW Document]. https://apps.ecology.wa.gov/publications/documents/1003034.pdf accessed 7.30.21.

[R129] Winchell LJ, Wells MJM, Ross JJ, Fonoll X, Norton JW, Kuplicki S, Khan M, Bell KY, 2021. Analyses of per- and polyfluoroalkyl substances (PFAS) through the urban water cycle: toward achieving an integrated analytical workflow across aqueous, solid, and gaseous matrices in water and wastewater treatment. Sci. Total Environ 774, 145257. 10.1016/J.SCITOTENV.2021.145257.

[R130] Winquist A, Lally C, Shin HM, Steenland K, 2013. Design, methods, and population for a study of PFOA health effects among highly exposed Mid-Ohio Valley community residents and workers. Environ. Health Perspect 121, 893–899. 10.1289/EHP.1206450. 23735518 PMC3734501

[R131] Xiao F, 2017. Emerging poly- and perfluoroalkyl substances in the aquatic environment: a review of current literature. Water Res. 124, 482–495. 10.1016/J.WATRES.2017.07.024. 28800519

[R132] Yao J, Pan Y, Sheng N, Su Z, Guo Y, Wang J, Dai J, 2020. Novel perfluoroalkyl ether carboxylic acids (PFECAs) and sulfonic acids (PFESAs): occurrence and association with serum biochemical parameters in residents living near a fluorochemical plant in China. Environ. Sci. Technol 54, 13389–13398. 10.1021/ACS.EST.0C02888. 33047597

[R133] Ye F, Tokumura M, Islam MS, Zushi Y, Oh J, Masunaga S, 2014. Spatial distribution and importance of potential perfluoroalkyl acid precursors in urban rivers and sewage treatment plant effluent – Case study of Tama RiverJapan. 67, 77–85. 10.1016/J.WATRES.2014.09.014. 25262552

[R134] Yeung LWY, Yamashita N, Taniyasu S, Lam PKS, Sinha RK, Borole DV, Kannan K, 2009. A survey of perfluorinated compounds in surface water and biota including dolphins from the Ganges River and in other waterbodies in India. Chemosphere 76, 55–62. 10.1016/J.CHEMOSPHERE.2009.02.055. 19328521

[R135] Young CJ, Furdui VI, Franklin J, Koerner RM, Derek, Muir DCG, Mabury SA, 2007. Perfluorinated acids in arctic snow: new evidence for atmospheric formation. Environ. Sci. Technol 41, 3455–3461. 10.1021/ES06262342012. 17547163

[R136] Zhang W, Zhang D, Liang Y, 2019. Nanotechnology in remediation of water contaminated by poly- and perfluoroalkyl substances: a review. Environ. Pollut 247, 266–276. 10.1016/J.ENVPOL.2019.01.045. 30685667

[R137] Zhang W, Cao H, Liang Y, 2021. Plant uptake and soil fractionation of five ether-PFAS in plant-soil systems. Sci. Total Environ 771, 144805. 10.1016/J.SCITOTENV.2020.144805. 33529820

[R138] Zhang Z, Sarkar D, Datta R, Deng Y, 2021. Adsorption of perfluorooctanoic acid (PFOA) and perfluorooctanesulfonic acid (PFOS) by aluminum-based drinking water treatment residuals. J. Hazard. Mater. Lett 2, 100034. 10.1016/J.HAZL.2021.100034.

