# Supplementary material for: Per- and polyfluoroalkyl substances in water and wastewater: A critical review of their global occurrence and distribution
Source: Sci Total Environ. Author manuscript; Available in PMC 2023 May 15. (PMC10184764; doi:10.1016/j.scitotenv.2021.151003)
Supplement: Supplementary Material [file NIHMS1853892-supplement-Supplementary_Material.docx]

**Per- and Polyfluoroalkyl Substances in Water and Wastewater – A Critical Review of their Global Occurrence and Distribution**

Sudarshan Kurwadkar^1,2*^, Jason Dane^1^, Sushil R. Kanel^3,4^, Mallikarjuna N. Nadagouda^5^, Ryan W. Cawdrey^1^, Balram Ambade^6^, Garrett C. Struckhoff^1^, Richard Wilkin^2**^

^1^Department of Civil and Environmental Engineering, California State University, 800 N. State College Blvd., Fullerton, CA 92831, USA

^2^Center for Environmental Solutions and Emergency Response, U.S. Environmental Protection Agency, 919 Kerr Research Drive, Ada, Oklahoma 74820, USA.

^3^Department of Chemistry, Wright State University, 3640 Colonel Glen Highway, Dayton, OH 45435

^4^Pegasus Technical Services, Inc., 46 E. Hollister Street, Cincinnati, OH 45219, USA

^5^Center for Environmental Solutions and Emergency Response, U.S. Environmental Protection Agency, 26 West Martin Luther King Drive, Cincinnati, Ohio 45268, USA.

^6^Department of Chemistry, National Institute of Technology, Jamshedpur 831014, Jharkhand, India

^*^Corresponding authors.

^*^(Dr. Sudarshan Kurwadkar)

Tel.: +1 (657) 278 2457

*E-mail address*: [skurwadkar@fullerton.edu](mailto:skurwadkar@fullerton.edu)

[kurwadkar.sudarshan@epa.gov](mailto:kurwadkar.sudarshan@epa.gov)

and

^**^(Dr. Richard Wilkin)

Tel.: +1 (580) 436-8874

*E-mail address*: [wilkin.rick@epa.gov](mailto:wilkin.rick@epa.gov)

**SUPPLEMENTARY INFORMATION**

The supplementary information presents the chemical properties of PFAS and worldwide occurrence of PFAS compounds in aquatic resources

**Table S1**

Chemical Properties of PFOA and PFOS

| **Property** | **PFOS** | **PFOA** |
| --- | --- | --- |
| Formula | C_8_HF_17_O_3_S | C_8_HF_15_O_2_ |
| Melting point  (°C) | 90 | 54 |
| Density (g cm^-3^) | 1.25 | 1.8 |
| Molecular weight (g mol^-1^) | 500 | 414 |
| Boiling Point (^0^C) | 258-260 | 192 |
| Vapor Pressure ( mm Hg at 25^0^ C) | 0.002 | 0.525 |
| Henry Law Constant at 25 ^0^C (Unitless) | NM* | 0.225 |
| Log Kow | NM* | 2.13 |
| Log Koc | 2.57 | 2.06 |
| Solubility (mg L^-1^) | 680 | 1780 |
| Appearance | White powder | Colorless liquid |
|  |  |  |

(**NM: Not Measurable, Source:* (USEPA, 2017)

**Table S2:** Distribution of PFOA and PFAS in various environmental matrices in the Asia Pacific Region

| Country | Media | PFOA (ngL^−1^) | PFOS (ngL^−1^) | Source |
| --- | --- | --- | --- | --- |
| China | Surface Water |  | 47 | UNEP, 2017 |
| China | Groundwater | 524 |  |  |
| China | Drinking water | 2.7 |  | Bao et al., 2011 |
| China (Huangpu River) | Surface Water | 1590 | 20.5 | Liu et al., 2012 |
| China (Shanghai) | Surface Water | 152 |  |  |
| China (Zhejiang Province) | Surface Water | 90 | 5 |  |
| China (Shanghai Province) | Surface Water | 182 |  |  |
| China (Huangpu River) | Surface Water | 105 | 5.4 | Lu et al., 2015 |
| China (Middle Yangtze River) | Surface Water | 297.5 | 37.8 | Wang et al., 2015 |
| China (Pearl River) | Surface Water | 13 | 99 |  |
| China (Lower Yangtze River) | Surface Water | 260 | 14 |  |
| China (Pearl River) | Surface Water | 8.7 | 11 | Liu et al., 2018 |
| China (Liaohe River) | Surface Water | 0.33 | 10.9 | Dong et al., 2018 |
| China (Guanting Reservoir) | Surface Water | 2.3 |  | Wang et al., 2011 |
| China (Remote areas) | Wastewater | 0.4 | 2.4 | Jin et al., 2009 |
| China (Yangtze River) | Surface Water | 36.5 | 12.12 | Pan et al. (2018) |
| China (Yellow River) | Surface Water | 4.92 | 4.4 |  |
| China (Pearl River) | Surface Water | 52.8 | 23.57 |  |
| China (Liao River) | Surface Water | 12.3 | 5.66 |  |
| China (Huai River) | Surface Water | 9.06 | 3.72 |  |
| China (Chao Lake) | Surface Water | 10.5 | 29.7 |  |
| China (Tai Lake) | Surface Water | 44.5 | 15.2 |  |
| China (Pearl River) | Surface Water | 13 | 99 | So et al. (2007) |
| China (Yangtze River) | Surface Water | 260 | 14 |  |
| China (Bohai Sea) | Surface Water | 845 | 2.91 | Zhao et al. 2020 |
| China (River Mounth Bohai Sea) | Surface Water | 7335 | 71.8 |  |
| China (Jiulong Estuary)-Dry Season | Surface Water | 14.74 | 20.8 | Wang et al. 2020 |
| China (Jiulong Estuary)Wet Season | Surface Water | 0.25 | 1.13 |  |
| China (Jiulong Estuary)-Wet Season | Bottom Water | 0.15 | 0.88 | Wang et al. 2020 |
| China (Multiple) | Tap Water | 26.33 | 2.8 | Li et al. 2019 |
| China (Beijing) | Precipitation | 13 | 0.62 | Johansson et al. 2018 |
| China (Wuhan) | Precipitation | 9.53 | 0.64 |  |
| Hongkong, China | Coastal water | **16 pg/L** | **12 pg/L** | So et al. (2004) |
| Australia | Groundwater | 1,800 | 5,560 | Gallen et al., 2018 |
| Australia | Groundwater | 2.2 | 34 | Szabo et al., 2018 |
| Australia (Oakey) | Groundwater | 600 | 14000 | Bräunig et al. 2017 |
| Australia (Oakey) | Surface Water | 120 | 940 | Marchiandi et al. 2021 |
| Australia (Various locations) | Drinking water | 9.7 | 16 | Thompson et al., 2011 |
| Tibet (Mt. Muztagata) | Snow and ice cores | 243 pgL^-1^ | 346 pgL^-1^ | Wang et al., 2014 |
| Tibet (Mt. Zuoqiupo) | Snow and ice cores | 183 pgL^-1^ | ND | Wang et al., 2014 |
| Japan | Surface Water |  | 230 | UNEP, 2018 |
| Japan (Tokyo Bay, Japan | Surface Water |  | 59 | Boulanger et al., 2004 |
| Japan (Osaka Bay, Japan | Surface Water |  | 21 |  |
| Japan (Lake Biwa, Japan | Surface Water |  | 7.4 |  |
| Japan (Ariake Bay, Japan | Surface Water |  | 11 |  |
| Japan (Tama River, Japan | Surface Water |  | 157 |  |
| Japan (Kamo River) | Surface Water | 36 | 4.1 | Senthilkumar et al., 2007 |
| Japan (Uji River-Station-I) | Surface Water | 100 | 8.7 |  |
| Japan (Uji River-Station-II) | Surface Water | 110 | 10 |  |
| Japan (Tenjin River) | Surface Water | 39 | 4.7 |  |
| Japan (Katsura River) | Surface Water | 7.9 | 2.6 |  |
| South Korea (Han River) | Surface Water | 4.53 | 3.86 | Pan et al. (2018) |
| South Korea | Surface Water |  | 33 | UNEP, 2019 |
| South Korea (Tama River) | Surface Water | 5.7 | 8 | Ye et al. (2014) |
| South Korea | Wastewater effluent | 12 | 31 |  |
| South Korea | Surface Water | <50 | <50 | Choi et al. (2017) |
| South Korea (Bukhan River) | Surface Water | 1.41 | 1.84 | Lam et al. (2014) |
| South Korea (Namhan River) | Surface Water | 0.64 | 6.25 |  |
| South Korea (Nakdong River) | Surface Water | 8.34 | 8.46 |  |
| South Korea (Nam River) | Surface Water | 4.65 | 1.06 |  |
| South Korea (Sangsa Lake) | Surface Water | 0.63 | 0.99 |  |
| South Korea (Yeongsan River) | Surface Water | 4.66 | 15.07 |  |
| South Korea (All sites) | Surface Water | 8.34 | 15.07 |  |
| Fiji (Waimanu River) | Surface water |  | 1.1 | Fiedler et al. (2018) |
| Fiji (Waimanu River) | Surface Water | <0.05 | <0.025 | Baabish et al., 2021 |
| Phillipines | Surface Water |  | 4.2 | UNEP, 2020 |
| Thailand | Surface Water |  | 54 | UNEP, 2021 |
| Thailand (Phong River) | Surface Water | 8.8 | 1.1 | Lien et al., (2006a, 2006b) |
| Malaysia (Kora Kinabalu) | Surface Water | 3.2 | 3.4 |  |
| Indian Rivers/Lakes/Coastal Areas | Surface Water |  | 3.91 | Yeung et al. (2009) |
| Indian Rivers/Lakes/Coastal Areas | Aquatic Biota |  | 27.9 ng·g^-1^ |  |
| India (Cooum River) | Surface Water |  | 3.91 |  |
| India | Raw Sewage |  | 12 |  |
| India (Dolphins-Ganges River) | Dolphins |  | 27.9 ng·g^-1^ |  |
| India (Ganges River) | Surface Water | 1.18 | 1.73 | Sharma et al. (2016) |
| Indian Ocean | Surface/Sew Water | 0.025 | 0.101 | González-Gaya et al. 2014 |
| Singapore (Urban Reservior) | Surface Water | 58 | 31 | Nguyen et al. (2016) |
| Singapore (Marina Catchment and Reservoir) | Surface Water | 37 | 20 | Nguyen et al. (2012) |
| Vietnam (Urban River Water) | Surface Water | 18 | 5.3 | Duong et al. (2015) |
| Vietnam | Groundwater | 4.5 | 8.2 |  |
| Vietnam (Red River) | Surface Water | 0.52 | 0.21 |  |
| Vietnam (Downstream from sewage discharge) | Surface Water | 53.5 | 40.2 | Lam et al. (2017) |
| Vietnam | Surface Water | 2.3 | 0.5 |  |
| Vietnam | Tap Water | 0.14 | ND |  |
| Vietnam | Ground Water | 1.93 | 0.32 |  |
| Vietnam (River Mekong, Do Quan Bridge) | Surface Water | 0.17 | 0.2 | Baabish et al., 2021 |
| Vietnam (Hanoi) | Surface Water | 6.6 | 1.3 | Lien et al., (2006a, 2006b) |
| Hongkong, China | Surface Water | 13.3 | 12 | Loi et al., 2011 |
| Mongolia (Tuul River) | Surface Water | 0.28 | 0.12 | Baabish et al., 2021 |
| Kiribati | Surface Water | 0.13 | 1.68 |  |
| Palau | Surface Water | 0.05 | 0.04 |  |
| Samoa (Vaisigano River - Lelata Point) | Surface Water | 0.05 | 0.05 |  |
| Solomon Islands | Surface Water | 0.14 | 1.12 |  |
| Tuvalu (Fongafale Islet) | Surface Water | 0.07 | 0.03 |  |
| Vanuatu | Surface Water | 0.84 | 6.23 |  |
|  |  |  |  |  |

**Table S3:** Distribution of PFOA and PFAS in various environmental matrices in the North American Region

| Country | Media | PFOA (ngL^-1^) | PFOS (ngL^-1^) | Source |
| --- | --- | --- | --- | --- |
| USA (Lake Ontario) | Surface Water | 70 | 70 | Boulanger et al., 2004 |
| USA (Lake Erie) | Surface Water | 524 |  |  |
| USA (Truckee River) | Surface Water | 6.90 | 2.20 | Bai & Son 2021 |
| USA (Las Vegas Wash) | Surface Water | 27.30 | 12.90 |  |
| Atlantic Ocean | Surface/Sea Water | 0.045 | 0.191 | González-Gaya et al. 2014 |
| Pacific Ocean | Surface/Sea Water | 0.023 | 0.0885 |  |
| Central to Eastern Pacific Ocean | Surface/Sea Water | 0.062 | 0.02 | Yamashita et al., 2005 |
| Western Pacific Ocean | Surface/Sea Water | 0.142 | 0.078 |  |
| North Atlantic Ocean | Surface/Sea Water | 0.338 | 0.036 |  |
| Mid Atlantic Ocean | Surface/Sea Water | 0.439 | 0.073 |  |
| USA (Little Hocking) | Drinking water | 1.5-7.2 |  | Bartell et al., 2010 |
| USA (Delaware River) | Surface Water | 5.7 | 6.92 | Pan et al. (2018) |
| USA (Washington State) | Surface Water | 95.6 | 7.6 | WDOE, 2017 |
| USA (Wurtsmith, Michigan) | Groundwater | 105,000 | 110,000 | (Moody et al., 2003 |
| USA (New Jersey) | Surface Water | 100 |  | NJDEP, 2014 |
| USA (New Jersey) | Groundwater |  | 57 |  |
| USA (Minnesota) | Groundwater | 42,000 | 2,700 | Oliaei et al., 2013 |
| USA (Minnesota) | Wastewater | 78 | 81 |  |
| USA (Minnesota) | Groundwater | 110 | 1,600 |  |
| USA (Minnesota) | Landfill gas condensate | 84,000 | 30,000 |  |
| USA (Minnesota) | Groundwater | 1,836,000 | 324,000 | Appleman et al., 2014b |
| USA (Minnesota) | Surface Water | 18,200 | 3,600 | Espana et al., 2015 |
| USA (MN, D/S 3M Mississippi River) | Surface Water | 94 | 90 | Nakayama et al., 2010 |
| USA (MN, Upper Mississippi River) | Surface Water | 125 | 245 |  |
| USA (San Jose, California) | Recycled Water | 190 | 190 | Plumlee et al., 2008 |
| USA (San Jose, California) | Surface Water | 36 | 56 |  |
| USA (San Jose, California) | Groundwater | 18 | 87 |  |
| USA (Municipal Marina, Columbus, GA) | Surface Water | 27 | 83 | Boulanger et al., 2004 |
| USA (Martin County, Port St. Lucie, FL) | Surface Water |  | 138 |  |
| USA (Drain Pond, Port St. Lucie, FL) | Surface Water | 760 | 51000 |  |
| USA (Flint Creek, Decatur, AL | Surface Water | 63 | 114 |  |
| USA (Tennessee River, Decatur, AL) | Surface Water | 120 | 220 | Newton et al. 2017 |
| USA( Mobile River, Mobile, AL | Surface Water | 83 | 43 | Boulanger et al., 2004 |
| USA( Three Mile Creek, Mobile, AL | Surface Water |  | 39 |  |
| Canada (Etobicoke Creek, Toronto, Canada | Surface Water | 11000 | 2200000 |  |
| USA (Tennessee River, Decatur, AL | Surface Water | 598 | 144 |  |
| USA (Lake Michigan) | Surface Water | 2.96 | ND | Bradley et al. 2020 |
| USA (Chicago) | Tap Water | 14.00 | 4.24 |  |
| USA (Chicago) | Tap Water, Pre-distribution | 3.23 | ND |  |
| USA (Large Water System) | Surface Water | 31 | 77 | Crone et al. 2019 |
| Canada/USA (Lake Erie, Canada/USA | Surface Water | 47 | 39 | Boulanger et al., 2004 |
| Canada/USA (Lake Ontario, Canada/USA | Surface Water | 70 | 121 | Boulanger et al., 2004 |
| Canada (Lake Superior) | Surface Water | 0.26 | 0.65 | DeSilva et al. (2011) |
| Canada (Lake Huron) | Surface Water | 2.25 | 3.22 |  |
| Canada (Lake Michigan) | Surface Water | 2 | 4.1 |  |
| Canada (Lake Erie) | Surface Water | 2.84 | 5.46 |  |
| Canada (Lake Ontario) | Surface Water | 5.51 | 4.31 |  |
| Canada (Meretta Lake) | Surface Water | 17 |  | Lescord et al. 2015 |
| Canada (Resolute Lake) | Surface Water | 9.4 |  |  |
| Canada (Char Lake) | Surface Water | 0.62 |  |  |
| Canada (Small Lake) | Surface Water | 0.6 |  |  |
| Canada (North Lake) | Surface Water | 0.66 |  |  |
| Canada (9 Mile Lake) | Surface Water | 0.69 |  |  |
| Canada (Amituk Lake) | Surface Water | 0.230 | 0.021 | Muir et al. 2019 |
| USA & Canada (Great Lakes) | Surface water | 1.5 | 2.1 | Gewurtz et al. 2020 |
| USA & Canada (Great Lakes, Multiple) | Precipitation | 0.46 | 0.93 |  |
| Mexico (Ohuira Bay) | Surface Water | 0.49 | 1.07 | Baabish et al., 2021 |
|  |  |  |  |  |

Table S4: Distribution of PFOA and PFAS in various environmental matrices in the South America and the Caribbean Region

| Country | Media | PFOA (ng L^-1^) | PFOS (n gL^-1^) | Source |
| --- | --- | --- | --- | --- |
| Brazil (Pariba do Sul River) | Surface Water | 1.32 | 1.22 | Quinete et al. (2009) |
| Brazil (Rio De Janerio, Sao Paulo) | Drinking Water | 2.82 | 6.7 | Quinete et al. (2009) |
| Brazil (Porto Alegre Metropolitan area) | Tap Water | 16 | 16 | Schwanz et al. 2016 |
| Uruguay | Surface Water |  | <1.0 | Fiedler et al. (2018) |
| Argentina (Rio de la Plata) | Surface Water | 1.44 | 5.32 | Baabish et al., 2021 |
| Brazil (Amazon River) | Surface Water | 0.15 | 0.35 | Baabish et al., 2021 |
| Brazil (São Vicente channel) | Surface Water | 0.86 | 4.08 | Baabish et al., 2021 |
| Ecuador (Babahoyo river) | Surface Water | 0.26 | 0.89 | Baabish et al., 2021 |
| Jamaica (Hunts Bay River, Causeway Bridge) | Surface Water | 1.34 | 2.32 | Baabish et al., 2021 |
| Portugal (Corvo) | Precipitation | 1.73 | 1.26 | Johansson et al. 2018 |

Table S5: Distribution of PFOA and PFAS in various environmental matrices in the European Region

| Country | Media | PFOA (ng L^-1^) | PFOS (ng L^-1^) | Source |
| --- | --- | --- | --- | --- |
| Netherlands (Rotterdam, Nieuwe Maas) | Surface Water |  | 11 | Fiedler et al. (2018) |
| Netherlands (Kampen IJssel) | Surface Water |  | 9.9 |  |
| Netherlands (Various cities) | Tap Water | 8.6 |  | Ullah et al., 2011 |
| Norway (multiple water) | Drinking Water | 2.5 |  | Haug et al., 2010 |
| Atlantic Ocean | Surface/Sea Water | 0.045 | 0.191 | González-Gaya et al. 2014 |
| North Atlantic Ocean | Surface/Sea Water | 0.338 | 0.036 | Yamashita et al., 2005 |
| Mid Atlantic Ocean | Surface/Sea Water | 0.439 | 0.073 |  |
| Sweden (Malaren Lake) | Surface Water | 3.34 | 8.23 | Pan et al. (2018) |
| Sweden (Råö) | Precipitation | 1.3 | 1.99 | Johansson et al. 2018 |
| Sweden (Stockholm) | Precipitation | 1.38 | 0.6 |  |
| Sweden | Tap Water |  | 8.8 | Ullah et al., 2011 |
| Sweden | Drinking Water |  | 45 | Gyllenhammar et al., 2015 |
| London (Thames River) | Surface Water | 11.7 | 18.8 | Schwanz et al. 2016 |
| France (Various Cities) | Tap Water | 13 | 20.6 | Harrad et al. 2019 |
| France (Various Cities) | Drinking Water | 12 | 22 | Boiteux et al., 2012 |
| Spain (Catalonia) | Drinking Water | 2.4 | 1.8 | Domingo et al. 2012 |
| Spain (Tarragona Province) | Tap Water | 6.28 | 0.87 | Ericson et al., 2008 |
| Spain (Barcelona metropolitan area) | Tap Water | 14 | 40 | Harrad et al. 2019 |
| Spain (Ebro River Basin) | Surface Water | 68 | 2709 | Llorca et al., 2012 |
| Spain (Ebro Delta, Catalonia) | Surface Water | 8.7 | 4.3 | Pignotti et al., 2017 |
| Spain (Jucar Basin) | Surface Water | 52.2 | 128 | Campo et al., 2016 |
| Spain (Cantabrian Sea, North Spain) | Port Seawater | 0.31 | 6.57 | Gómez, et al., 2011 |
| Spain (Cantabrian Sea, North Spain) | Wastewater Effluent | 3.38 | 2.16 |  |
| Spain (Ebro River) | Surface Water | 125 | 27 | Lorenzo et al., 2016 |
| Spain (Guadalquivir River) | Surface Water | 188.6 | 42.6 |  |
| Spain (Llobregat River) | Surface Water | 146 | 27110 | Campo et al., 2015 |
| Spain (Llobregat River) | Surface Water | 5.5 | 21 | Flores et al., 2013 |
| Spain (Ebro River Basin) | Wastewater Effluent | 0.92 | 498 | Campo et al., 2014 |
| Spain (Guadalquivir River Basin) | Wastewater Effluent | 286 | 8.64 |  |
| Spain (Jucar River Basin) | Wastewater Effluent | 2390 | 403 |  |
| Spain (Llobregat River Basin) | Wastewater Effluent | 732 | 3100 |  |
| Spain (WWTP effluent) | Treated wastewater | 17 | 501 | Llorca et al., 2012 |
| Ireland (Tap Water Community) | Tap Water | 1.76 | 0.76 | Fiedler et al. (2018) |
| Ireland (Tap Water Private Supply) | Tap Water | 1.3 | <0.15 |  |
| Turkey | Tap Water | 0.4 | 0.52 | Endirlik et al. 2019 |
| Germany (Lake Constance) | Surface Water | 1 | 3 | Lange et al., 2007 |
| Germany (Sauerland) | Drinking Water | 640 |  | Domingo et al. 2017 |
| Germany (German Bight) | Surface Water | 0.71 | 0.157 | Joerss et al. 2019 |
| Germany (Elber River) | Surface Water | 2.1 | 0.61 | Joerss et al. 2019 |
| Germany (Baltic Sea) | Surface Water | 0.7 | 0.18 |  |
| Germany (Oder Lagoon) | Surface Water | 0.79-1.1 | 0.26 |  |
| Germany (Rivers, White Elster, Saale &Elbe) | Surface Water | 26.53 | 3 | Shafique et al.2017 |
| Germany (Leipzig) | Tap Water | 6.15 |  |  |
| Germany | Surface Water | 6.5 | 4.6 | Llorca et al., 2012 |
| Germany (WWTP effluent) | Treated wastewater | 18 | 0.22 |  |
| Germany (Rhine River) | Surface Water | 3.66 | 8.56 | Pan et al. (2018) |
| Germany (Rhine River) | Surface Water | 9 | 26 | Skutlarek et al. (2006a) |
| Germany (Ruhr, Emscher, Lippe) | Surface Water | 48 | 18 |  |
| Germany (Rhine-Herne Canal) | Surface Water | 34 |  |  |
| Germany (Rhine-Wesel-Datteln Canal) | Surface Water | 36 |  |  |
| Germany (Lake Mlehne) | Surface Water | 654 | 17 |  |
| Germany (Oberhausen) | Drinking Water | 43 | 9 |  |
| Germany (Mulheim) | Drinking Water | 30 | 3 |  |
| Germany (Velbert) | Drinking Water | 38 |  |  |
| Germany (Essen) | Drinking Water | 56 | 7 |  |
| Germany (Bochum) | Drinking Water | 53 | 10 |  |
| Germany (Witten) | Drinking Water | 49 | 12 |  |
| Germany (Hagen) | Drinking Water | 34 | 22 |  |
| Germany (Dortmund) | Drinking Water | 152 | 11 |  |
| Germany (Schwerte) | Drinking Water | 145 | 13 |  |
| Germany (Froendenberg) | Drinking Water | 143 | 6 |  |
| Germany (Menden) | Drinking Water | 157 | 11 |  |
| Germany (Wickede) | Drinking Water | 208 |  |  |
| Germany (Neheim) | Drinking Water | 519 | 5 |  |
| Germany (Arnsberg) | Drinking Water | 71 |  |  |
| Germany (Meschede) | Drinking Water | 22 |  |  |
| Germany (Berlin) | Drinking Water | 2 | 6 |  |
| Germany (Koblenz) | Drinking Water | 2 | 5 |  |
| Germany (Muenster) | Drinking Water | 4 |  |  |
| Germany (Ruhr (Duisburg)) | Surface Water | 48 | 5 |  |
| Germany (Ruhr (Muelheim)) | Surface Water | 46 | 6 |  |
| Germany (Ruhr (Essen)) | Surface Water | 51 | 7 |  |
| Germany (Rhur (Bochum) | Surface Water | 57 | 4 |  |
| Germany (Ruhr(Witten) | Surface Water | 69 | 22 |  |
| Germany (Ruhr (Hagen) | Surface Water | 90 | 13 |  |
| Germany (Ruhr (Schwerte) | Surface Water | 177 | 14 |  |
| Germany (Ruhr (Huesten) | Surface Water | 63 |  |  |
| Germany (Ruhr (Meschede-Wennemen)) | Surface Water | 24 |  |  |
| Germany (Ruhr (Meschede-Wehrstapel)) | Surface Water | 139 |  |  |
| Germany (Moehne (Neheim) | Surface Water | 647 | 14 |  |
| Germany (Lake Moehne (Westrich)) | Surface Water | 654 | 17 |  |
| Germany (Moehne (Heidberg)) | Surface Water | 3640 | 193 |  |
| Germany (Moehne (Brilon)) | Surface Water | 11 |  |  |
| Germany (Elpe (Bestwig)) | Surface Water | 1168 |  |  |
| Germany (Lenne (Hagen)) | Surface Water | 59 | 11 |  |
| Germany (Rhine (Neuss)) | Surface Water |  | 5 |  |
| Germany (Rhine (Duisburg-Walsum) | Surface Water | 9 | 9 |  |
| Germany (Moehne (Klossiepen) | Surface Water | 11 |  |  |
| Germany (Moehne (Steinbecke) | Surface Water | 16800 | 5900 |  |
| Germany (Moehne (Klossiepen) | Surface Water | 1860 | 2 |  |
| Germany (Bermecke (mouth)) | Surface Water | 6530 | 507 |  |
| Germany (Moehne) | Surface Water | 1930 | 135 |  |
| Germany (Moehne (Steinbecke mouth) | Surface Water | 33900 | 3160 |  |
| Germany (Moehne) | Surface Water | 7070 | 405 |  |
| Germany (Moehne) | Surface Water | 5990 | 370 |  |
| Germany (Moehne) | Surface Water | 5570 | 311 |  |

Table S6: Distribution of PFOA and PFAS in various environmental matrices in the African Region

| Country | Media | PFOA (ng L^-1^) | PFOS (ng L^-1^) | Source |
| --- | --- | --- | --- | --- |
| Mali (Sotuba/Mali) | Surface Water |  | 5.7 | Fiedler et al. (2018) |
| Ethiopia (Lake Tana) | Surface Water | 0.69 | 0.22 | Ahrens and Bundschuh, 2014b |
| Nigeria | Surface Water |  | 1.39 | UNEP 2015b |
| Morocco | Surface Water |  | 0.035 |  |
| Congo | Surface Water |  | 0.035 |  |
| Uganda (Lake Victoria Basin, Open Lake) | Surface Water | 1.46 | 0.03 | Arinaitwe et al. (2021) |
| Uganda (Lake Victoria Basin, Murchison Bay) | Surface Water | 4.88 | 1.29 |  |
| Uganda (River Kagera) | Surface Water | 3.64 | 0.06 |  |
| Uganda (River Sio) | Surface Water | 3.35 | 0.14 |  |
| Uganda (Nakivubo Channel) | Surface Water | 9.05 | 7.02 |  |
| Uganda (Kampala) | Tap Water | 2.71 | 0.40 |  |
| Uganda (Jinja) | Tap Water | 3.56 | 0.05 |  |
| Uganda (Entebbe) | Tap Water | 1.96 | 0.06 |  |
| Uganda (Lake Victoria, Nakivubo Channel ) | Surface Water | 2.4 | 1.6 | Dalahmeh et al. 2018 |
| Uganda (Lake Victoria, Nakivubo Channel ) | Surface Water | 3.9 | 3.9 |  |
| Uganda (Lake Victoria, Ggabba, Murchison Bay) | Surface Water | 0.6 | <LOD |  |
| Uganda (Lake Victoria, Ggabba, Murchison Bay) | Surface Water | <LOD | <LOD |  |
| Uganda (Lake Victoria Basin) | Precipitation | 0.63 | 0.11 | Arinaitwe et al. (2021) |
| South Africa (Vaal River) | Surface Water | 4.6 | 35.7 | Groffen et al. 2018 |
| Kenya (River Sosiani) | Surface Water | 9.99 | ND | Shafique et al.2017 |
| Kenya (Winam Gulf) | Groundwater | 11.7 | 2.53 | Orata et al. 2008 |
| Kenya (Winam Gulf) | Surface Water | 96.4 | 13.23 |  |
| Kenya (Sabaki River Mouth) | Surface Water |  | 4.6 | Fiedler et al. (2018) |
| Kenya (Sabaki River) | Surface Water | 2.13 | 1.94 | Baabish et al., 2021 |
| Egypt (River Nile) | Surface Water | 0.72 | 0.38 |  |
| Ghana (Volta River) | Surface Water | 0.23 | 0.23 |  |
| Senegal (River Senegal) | Surface Water | 0.13 | 0.36 |  |
| Tunisia (Oued Medjerda) | Surface Water | 1.6 | 0.94 |  |
| Zambia (Kafue/Zambezi Confluence) | Surface Water | 0.14 | 0.14 |  |

**References:**

Appleman TD, Higgins CP, Quiñones O, Vanderford BJ, Kolstad C, Zeigler-Holady JC, et al. Treatment of poly- and perfluoroalkyl substances in U.S. full-scale water treatment systems. Water Research 2014b; 51: 246-255.

Arinaitwe K, Keltscha N, Taabu-Munyaho A, Reemtsma T, Berger U. Perfluoroalkyl substances (PFASs) in the Ugandan waters of Lake Victoria: Spatial distribution, catchment release and public exposure risk via municipal water consumption. Science of the Total Environment 2021, 783: 146970

Bartell, S.M., Calafat, A.M., Lyu, C., Kato, K., Ryan, P.B., Steenland, K., 2010. Rate of decline in serum PFOA concentrations after granular activated carbon filtration at two public water systems in Ohio and West Virginia. Environ. Health Perspect. 118 (2), 222–228.

Boiteux, V., Dauchy, X., Rosin, C., Munoz, J.F., 2012. National screening study on 10 perfluorinated compounds in raw and treated tap water in France. Arch. Environ.

Contam. Toxicol. 63 (1), 1–12.

Bradley, P.M., Argos, M., Kolpin, D.W., Meppelink, S.M., Romanok, K.M., Smalling, K.L., et al., 2020. Mixed organic and inorganic tapwater exposures and potential effects in greater Chicago area, USA. Sci. Total Environ. 719, 137236.

Crone BC, Speth TF, Wahman DG, Smith SJ, Abulikemu G, Kleiner EJ, Pressman JG. 2019. Occurrence of per‐ and polyfluoroalkyl substances (PFAS) in source water and their treatment in drinking water. Crit Rev Environ Sci Technol 49(24):2359–2396.

Dalahmeh S, Tirgani S, Komakech AJ, Niwagaba CB, Ahrens L. Per- and polyfluoroalkyl substances (PFASs) in water, soil and plants in wetlands and agricultural areas in Kampala, Uganda. Sci. Total Environ. 2018; 631–632: 660–667.

Dong W, Liu B, Song Y, Zhang H, Li J, Cui X. Occurrence and Partition of Perfluorinated Compounds (PFCs) in Water and Sediment from the Songhua River, China. Archives of Environmental Contamination and Toxicology 2018; 74: 492-501.

Endirlik, Ü. B., Bakır, E., Boşgelmez, İ.İ., Eken, A., Narin, İ., Gürbay, A., 2019. Assessmentof perfluoroalkyl substances levels in tap and bottled water samples from Turkey. Chemosphere 235, 1162–1171.

Ericson, I., Nadal, M., van Bavel, B., Lindström, G., Domingo, J.L., 2008a. Levels of perfluorochemicals in water samples from Catalonia, Spain: is drinking water a significant contribution to human exposure? Environ. Sci. Pollut. Res. Int. 15 (7), 614–619.

Fiedler, H., Kennedy, T., Henry, B. J. A Critical Review of a Recommended Analytical and Classification Approach for Organic Fluorinated Compounds with an Emphasis on Per‐ and Polyfluoroalkyl Substances. Integrated Environmental Assessment and Management, 2019, 17(2), 331-351

Flores, C., Ventura, F., Martin-Alonso, J., Caixach, J., 2013. Occurrence of perfluorooctane sulfonate (PFOS) and perfluorooctanoate (PFOA) in N.E. Spanish surface waters and their removal in a drinking water treatment plant that combines conventional and advanced treatments in parallel lines. Sci. Total Environ. 461–462, 618–626.

Gómez C, Vicente J, Echavarri-Erasun B, Porte C, Lacorte S. Occurrence of perfluorinated compounds in water, sediment and mussels from the Cantabrian Sea (North Spain). Marine Pollution Bulletin, 2011, 62, 948-955

González-Gaya, B., Dachs, J., Roscales, J.L., Caballero, G., Jiménez, B., Perfluoroalkylated substances in the global tropical and subtropical surface oceans. Environ. Sci. Technol. 2014, 48, 13076–13084.

Gyllenhammar, I., Berger, U., Sundström, M., McCleaf, P., Eurén, K., Eriksson, S., Ahlgren, S., Lignell, S., Aune, M., Kotova, N., Glynn, A., 2015. Influence of contaminated drinking water on perfluoroalkyl acid levels in human serum–A case study from Uppsala, Sweden. Environ. Res. 140, 673–683.

Haug, L.S., Salihovic, S., Jogsten, I.E., Thomsen, C., van Bavel, B., Lindström, G., Becher,

G., 2010. Levels in food and beverages and daily intake of perfluorinated compounds in Norway. Chemosphere 80 (10), 1137–1143.

Joerss, H., Apel, C., Ebinghaus, R., 2019. Emerging per- and polyfluoroalkyl substances (PFASs) in surfacewater and sediment of the north and Baltic seas. Sci. Total Environ. 686, 360–369.

Johansson, J.H., Shi, Y., Salter, M., Cousins, I.T., 2018. Spatial variation in the atmospheric deposition of perfluoroalkyl acids: source elucidation through analysis of isomer patterns. Environ Sci Process Impacts 20, 997–1006.

Lange, F.T., Wenz, M., Schmidt, C.K., Brauch, H.J., 2007. Occurrence of perfluoroalkyl sulfonates and carboxylates in German drinking water sources compared to other countries. Water Sci. Technol. 56 (11), 151–158.

Lescord, G.L., Kidd, K.A., De Silva, A.O., Williamson, M., Spencer, C., Wang, X., Muir, D.C.G., 2015. Perfluorinated and polyfluorinated compounds in lake food webs from the Canadian high arctic. Environ. Sci. Technol. 49, 2694e2702. <https://10.1021/es5048649>.

Liu W, Dong G, Luo Y, Liu L, Cao Z, Li X, et al. Estimation of Reference Values for PFOS and PFOA in Human Biomonitoring and Relevance of Exposure among Family Members in China. Journal of Environmental Protection 2012; 3: 353-361.

Nakayama, S. F., Strynar, M. J., Reiner, J. L., Delinsky, A. D., Lindstrom, A. B. Determination of Perfluorinated Compounds in the Upper Mississippi River Basin. Environ. Sci. Technol 2010, 44, 4103-4109

Orata F, Quinete N, Maes A, Werres F, Wilken R-D. Perfluorooctanoic acid and perfluorooctane sulfonate in Nile perch and tilapia from gulf of Lake Victoria. Afr. J. Pure Appl. Chem. 2008; 2: 075–079.

Senthilkumar, K.O., Ohi, E., Sajwan, K., Takasuga, T., Kannan, K., 2007. Perﬂuorinated compounds in river water, river sediment, market ﬁsh, and wildlife samples from Japan. Bull. Environ. Contam. Toxicol. 79 (4), 427e431.

Shafique, U., Schulze, S., Slawik, C., Böhme, A., Paschke, A., Schüürmann, G., 2017. Perfluoroalkyl acids in aqueous samples from Germany and Kenya. Environ. Sci. Pollut. Res. 24, 11031–11043.

Skutlarek, D., Exner, M., Farber, H., 2006. Perfluorinated surfactants in surface and drinking waters. Environ. Sci. Pollut. Res. Int. 13, 299. https://doi.org/10.1065/espr2006.07.326.

Ullah, S., Alsberg, T., Berger, U., 2011. Simultaneous determination of perfluoroalkyl phosphonates, carboxylates, and sulfonates in drinking water. J. Chromatogr. A 1218

(37), 6388–6395.

Yamashita, N., Kannan, K., Taniyasu, S., Horii, Y., Petrick, G., Gamo, T., 2005. A global survey of perfluorinated acids in oceans. Marine Pollution Bulletin 51, 658–668.
